# Siderophore-metal complexes in *Paraburkholderia phymatum*: structure elucidation of phymabactin

**DOI:** 10.1007/s10534-026-00823-9

**Published:** 2026-05-04

**Authors:** Luca Bürgi, Daphné Golaz, Gabriella Pessi, Laurent Bigler

**Affiliations:** 1 Department of Chemistry, University of Zurich, Zurich, Switzerland; 2 Department of Plant and Microbial Biology, University of Zurich, Zurich, Switzerland

**Keywords:** Rhizobia, Siderophore, UHPLC-HRMS, MS/MS fragmentation, Ion mobility, NMR

## Abstract

The symbiotic interaction between rhizobia and host plants takes place inside root nodules. Besides reducing atmospheric nitrogen into ammonium, which is then used by the plant to grow in nitrogen-deficient soils, certain rhizobia produce siderophores which enable iron uptake from the soil. Siderophores are strong iron chelators due to their hydroxamate, catecholate or carboxylate functional groups. In this work, the siderophores of the beta-rhizobial genus *Paraburkholderia* were analyzed by ultra-high-performance liquid chromatography (UHPLC) coupled to high-resolution mass spectrometry (HRMS). In particular, the production of the novel siderophore phymabactin by *Paraburkholderia phymatum* was confirmed and the structures of eleven derivatives were elucidated by tandem mass spectrometry (MS/MS) and nuclear magnetic resonance (NMR) spectroscopy. Phymabactins, consisting of a linear tetrapeptide backbone with hydroxamate and hydroxy-carboxylate groups, are structurally closely related to an already described family of siderophores called ornibactins. The acyl chain found in phymabactin derivatives is made up of eight, ten or twelve carbon atoms, contrarily to the more hydrophilic ornibactin derivatives with shorter acyl chains. Interestingly, spontaneous metal complexation of phymabactin derivatives with aluminum was observed. To explore this complexation in more detail, the collision cross sections (CCS) of phymabactins, ornibactins and corresponding metal complexes were determined by trapped ion mobility spectrometry. The CCS values of aluminum-phymabactin complexes were smaller than those of iron-phymabactin complexes, while an inverse relationship was observed for ornibactin complexes. In summary, this study discloses the molecular structures of phymabactins and investigates their metal complex formation.

**Supplementary Information:**

The online version contains supplementary material available at https://doi.org/10.1007/s10534-026-00823-9.

## Introduction

The growth of bacteria requires iron, which is the fourth most abundant element in the Earth’s crust (Yaroshevsky 2006). Despite its abundance, ferric iron (Fe^3+^) is nearly insoluble in aerobic environments at physiological pH and thus not easily bioavailable for bacteria (Hider and Kong 2010; Kümmerli 2023). A strategy of bacteria to solubilize Fe^3+^ is the secretion of small organic chelators with high iron-affinity, so-called siderophores (Gomes et al. 2024; Vraspir and Butler 2009). Specific receptor proteins transport the iron-siderophore complex into the cytoplasm, where the reduced ferrous iron (Fe^2+^) is released from the siderophore (Gomes et al. 2024; Hider and Kong 2010). Fe^2+^ is well soluble in aqueous systems at physiological pH and is thus accessible to bacteria and other microorganisms (Saha et al. 2016). In addition to iron, siderophores were reported to chelate other metal ions, such as Ag^+^, Al^3+^, Co^2+^, Cu^2+^, Ga^3+^ or Hg^2+^ (Braud et al. 2009; Mathew et al. 2016; Schalk et al. 2011). Another term frequently used for natural products that form coordination complexes with multiple metal ions is metallophores (de Kruijff et al. 2025). For instance, the hydroxamate metallophore, deferoxamine B, chelates Al^3+^ and is therefore used to treat aluminum intoxications (Bellotti and Remelli 2021; Saha et al. 2016). Aluminum is the third most abundant element in the Earth’s crust, one place ahead of iron (Yaroshevsky 2006), and has no known biological function (Schalk et al. 2011). Concerns arise due to its increased mobilization in acidic environments, which can lead to accumulation in plants and neurotoxic effects in human (Alasfar and Isaifan 2021). Siderophores are therefore of increasing interest for application in the bioremediation of heavy metal contaminated environments (Khan et al. 2018). The versatile applications of siderophores in chelation therapy, crop protection or bioremediation explain why new structures are highly sought-after.

*Paraburkholderia phymatum* STM815^T^ is a diazotrophic bacterial legume symbiont, which was isolated for the first time in 2001 (Moulin et al. 2001; Vandamme et al. 2002) and whose genome is available since 2014 (Moulin et al. 2014). *P. phymatum* STM815^T^ is a motile, rod-shaped beta-proteobacterium (beta-rhizobia), which can establish a beneficial interaction with more than 50 different legume species and was proven to be highly competitive for colonizing plants’ roots compared to other members of the soil microbiota (Lardi et al. 2017). Rhizobia are soil bacteria capable of performing nitrogen fixation by converting atmospheric nitrogen into ammonia, which plants can use as a source of nitrogen. Rhizobia are estimated to contribute up to 60% of the biological nitrogen fixation worldwide, underscoring their key role in the nitrogen cycle (Stagnari et al. 2017). Rhizobia also have other beneficial effects on plants, such as providing them with other micronutrients (iron and phosphate, for instance) or, protecting them against pathogens with the help of siderophores (Dardanelli et al. 2010; Das et al. 2017). A gene cluster with the potential to produce a new type of siderophore, called phymabactin, was identified in the genome of *P. phymatum* (Esmaeel et al. 2016). Recently, a *P. phymatum* mutant lacking the phymabactin gene cluster (*phm*) was shown to lose its siderophore activity, which was determined quantitatively using the chrome azurol S (CAS) assay (Golaz et al. 2024). The siderophore phymabactin was also observed to bind to aluminum and to be important in enabling *P. phymatum*’s survival in culture media supplemented with heavy metals such as zinc, copper or aluminum (Golaz et al. 2025). Another *Paraburkholderia* species, *Paraburkholderia tuberum,* cannot produce siderophores, despite sharing similarities with *P. phymatum*, such as the ability to form a symbiosis with tropical legumes (Vandamme et al. 2002). However, integration of the gene cluster coding for the siderophore ornibactin allowed *P. tuberum* to be more resistant to heavy metal induced stress, suggesting that siderophores facilitate resiliency against metal toxicity in plant-beneficial soil bacteria (Mathew 2015).

**Fig. 1:**
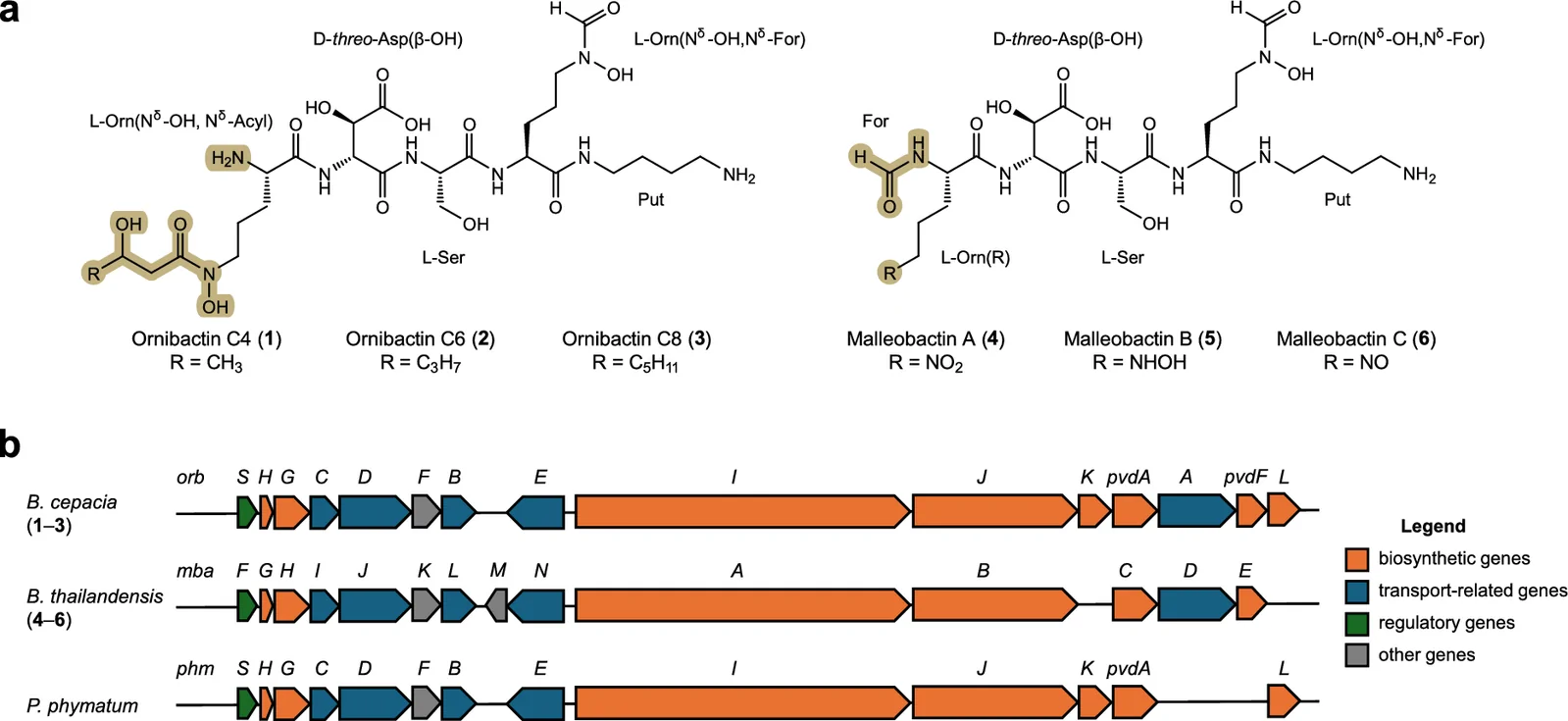
**a** Structures of ornibactin derivatives (**1**‒**3**) isolated from *Burkholderia cepacia* (Stephan et al. 1993), and malleobactin derivatives (**4**‒**6**) isolated from *Burkholderia thailandensis* (Franke et al. 2013). Those siderophores consist of the building blocks ornithine (Orn), aspartic acid (Asp), serine (Ser) and putrescine (Put), which have been modified with acylation (Acyl), hydroxylation (OH) or formylation (For). **b** Comparison of the ornibactin, malleobactin and phymabactin biosynthetic gene clusters (*orb, mba* and *phm*, respectively)

Ornibactins and malleobactins are well-described families of mixed-type hydroxamate and carboxylate siderophores sharing an identical linear tetrapeptide backbone (Franke et al. 2013; Stephan et al. 1993). Stephan and co-workers elucidated three ornibactin derivatives (**1**‒**3**), which differ in their β-hydroxylated acyl chain consisting of four, six or eight carbon atoms (see Fig. 1a). A total of nine related structures within the malleobactin family have been reported (Franke et al. 2015; Picard et al. 2022). Three representative malleobactins (**4**‒**6**), containing unusual derivatives of the amino acid ornithine (Orn) with nitro, hydroxylamine or nitroso groups, are shown in Fig. 1a. The ornibactin, malleobactin and phymabactin biosynthetic gene clusters (*orb*, *mba* and *phm*, respectively) depicted in Fig. 1b exhibit a high degree of similarity. Each gene cluster contains two non-ribosomal peptide synthetase (NRPS) genes (*orbI* and *orbJ*, *mbaA* and *mbaB*, *phmI* and *phmJ*), which contain four modules each responsible for the incorporation of an amino acid (Franke et al. 2015; Golaz et al. 2024; Thomas 2007). Variations in the predicted substrate specificities of the first and fourth adenylation domains of the NRPS may contribute to the structural differences among the N- and C-terminal amino acids of ornibactins, malleobactins and phymabactins (Butt and Thomas 2017; Franke et al. 2015). The second and third adenylation domains are consistently predicted to accept Asp and Ser, respectively (Blin et al. 2023; Butt and Thomas 2017). Similarly to *orb* and unlike *mba*, *phm* contains genes that encode the acetyltransferases *phmK* and *phmL*. Furthermore, *orb* and *phm* lack *mbaM*, predicted to encode a protein of unknown function (Vargas-Straube et al. 2016). Finally, the formyl-transferases *pvdF* and *mbaE*, which convert Orn(N^δ^-OH) to Orn(N^δ^-OH, N^δ^-Formyl), are present in *orb* and *mba*, but absent from the *phm* gene cluster (Kenjic et al. 2019).

UHPLC coupled to HRMS has become the gold standard for separating metabolites in complex natural extracts based on their polarity and mass-to-charge ratio (*m*/*z*). Many organic and organometallic compounds are readily ionized by electrospray ionization (ESI), providing stoichiometric information at low limits of detection. However, the gas-phase ions generated by ESI do not necessarily reflect the metal-ligand solution equilibria, which makes it challenging to determine complex formation constants by ESI-MS (Di Marco and Bombi 2006). Furthermore, MS/MS fragmentation spectra of selected precursor ions provide structural information and enable metabolite identification (Wolfender et al. 2015). Structure elucidation of unknown metabolites is frequently based on NMR spectra of the isolated compound in combination with MS/MS data, as reported for the siderophore albachelin produced by *Amycolatopsis alba* (Kodani et al. 2015). Recently, mass spectrometers have been complemented with ion mobility cells, which separate gas phase ions based on their size, shape and charge (Gabelica and Marklund 2018). In trapped ion mobility spectrometry (TIMS), for example, ions are separated using an electric field gradient and a drift gas pointing in opposite directions (Schroeder et al. 2019). Specific CCS values were derived for pyoverdines (Rehm et al. 2023b) and marine bacterial extracts (Bell et al. 2025) using TIMS, in the absence and presence of iron. In this work, the siderophores of the beta-rhizobia *P. phymatum* were analyzed by UHPLC-TIMS-HRMS. In particular, the production of the novel siderophore phymabactin by *P. phymatum* was confirmed and the structures of eleven derivatives were elucidated by MS/MS and NMR spectroscopy.

## Materials and methods

### Chemicals and reagents

UHPLC-MS grade acetonitrile (CH_3_CN), methanol (CH_3_OH), isopropanol and formic acid (HCOOH) were purchased from *Biosolve BV* (Valkenswaard, Netherlands). Ultrapure H_2_O (< 5 ppb TOC) was produced with a Milli-Q Advantage A10 water purification system (*Merck*, Bedford, MA, USA). Iron(III) chloride hexahydrate of 99% purity, aluminum(III) chloride hexahydrate of 99% purity and deuterium oxide (D_2_O) of 99.8% purity were purchased from *Sigma Aldrich* (St. Louis, MO, USA). ESI-L low concentration tuning mix was purchased from *Agilent* (Santa Clara, CA, USA). A sodium formate calibration solution (10 mM) was prepared by adding 1 M NaOH (250 µL) and HCOOH (50 µL) to isopropanol (12.5 mL) and ultrapure H_2_O (12.5 mL). The advanced Marfey’s reagent 1-fluoro-2,4-dinitrophenyl-5-D-leucine-*N,N*-dimethylethylenediamine-amide (D-FDLDA) was purchased from *Nacalai Tesque* (Kyoto, Japan). Racemic DL-*threo*-β-hydroxyaspartic acid was purchased from *Sigma Aldrich* (St. Louis, MO, USA) and enantiopure L-*threo*-β-hydroxyaspartic acid was purchased from *MedChemExpress* (Sollentuna, Sweden). Eppendorf Research plus pipettes (*Eppendorf*, Hamburg, Germany) were used. A CentriVap Refrigerated Centrifugal Concentrator from *Labconco* (Kansas City, MO, USA) was used for lyophilization.

### Bacterial strains, media and cultivation

*P. phymatum* STM815^T^ (Moulin et al. 2001) and *P. tuberum*::*orb* (Mathew 2015) were grown in LB-NaCl medium or AB minimal medium prepared without iron using 15 mM succinate (*Sigma Aldrich*) or 10 mM of D-glucose (*Sigma Aldrich*) as a carbon source, respectively (Clark and Maaløe 1967). To isolate the siderophores secreted by *P. phymatum* STM815^T^ and *P. tuberum*::*orb* cells, the bacteria were cultured in 250 mL Erlenmeyer flasks (starting OD_600_ = 0.05) at 180 rpm and 28 °C. After 17 h of incubation, the cells were precipitated (10′000 rpm, 10 min), the cell pellet was discarded and the supernatant was filter-sterilized with a 0.2 µm-pore filter (Filtropur 0.2, *Sarstedt*, Germany).

### Sample preparation for siderophore screening

Bacterial supernatants of *P. phymatum* and *P. tuberum*::*orb* were subjected to siderophore screening based on our published method (Rehm et al. 2023a). Therefore, 200 µL of bacterial supernatant was frozen using liquid nitrogen and lyophilized overnight. The dry lyophilizate was reconstituted in 300 µL of H_2_O/CH_3_OH (3:2) and centrifuged for 10 min at 5 °C and 14′000 rpm. Three vials were individually filled with 100 µL of supernatant. One vial was spiked with 1 µL of an aqueous 100 mM FeCl_3_ solution, another vial was spiked with 1 µL of an aqueous 100 mM AlCl_3_ solution, and the last vial was not spiked. These samples were analyzed in triplicate by UHPLC-TIMS-HRMS to investigate the formation of iron-siderophore and aluminum-siderophore complexes.

### UHPLC-TIMS-HRMS method

Samples were analyzed using a Vanquish Horizon UHPLC system (*Thermo Fisher*, Waltham, MA, USA) connected to a timsTOF Pro quadrupole-time-of-flight (QTOF) mass spectrometer (*Bruker*, Bremen, Germany) employing trapped ion mobility spectrometry (TIMS). The Vanquish Horizon UHPLC system was built from a binary pump H, a split sampler HT and a temperature-controlled column compartment.

Chromatographic separation was performed at 40 °C with an ACQUITY HSS T3 UPLC column (100 Å, 1.8 µm particle size, 2.1 × 100 mm, *Waters*, Milford, USA). The injection volume was 1 µL. The mobile phase consisted of A: H_2_O + 0.1% HCOOH and B: CH_3_CN + 0.1% HCOOH. A constant flow rate of 0.5 mL/min was applied using the following gradient: (I) 5% B isocratic from 0.0 to 0.5 min; (II) linear increase to 45% B from 0.5 to 6.0 min; (III) linear increase to 100% B from 6.0 to 6.1 min; (IV) 100% B isocratic from 6.1 to 9.0 min; (V) linear decrease to 5% B from 9.0 to 9.1 min; (VI) 5% B isocratic from 9.1 to 12.0 min. For internal mass and ion mobility calibration, a 1:1 mixture of 10 mM sodium formate and ESI-L low concentration tuning mix was injected at the beginning of each LC run using a syringe connected to a 6-port valve with a 20 µL loop.

The mass spectrometer was operated in the positive ESI mode at 4′500 V capillary voltage and 500 V endplate offset with a N_2_ nebulizer pressure of 2.2 bar and a dry gas flow of 10 L/min at 220 °C. TIMS inverse reduced mobility values ($1/{\mathrm{K}}_{0}$) were recorded in a range from 0.45 to 1.45 $1/{\mathrm{K}}_{0}$ with a ramp time of 100.0 ms, an ion charge control of 5 million and an ion funnel radiofrequency of 200 Vpp. N_2_ with a temperature of 305 K was used as drift gas. Ion mobility measurements were calibrated between 0.545 and 1.199 $1/{K}_{0}$ using an ESI-L low concentration tuning mix giving a mobility deviation below 0.4%. Mass spectra were acquired in a mass range from *m/z* 20 to 1′300 at a resolution of 40′000 (*m/z* 431 full width at half maximum). Mass calibration was performed between *m/z* 91 and 1′247 using a 10 mM solution of sodium formate giving a mass accuracy below 2 ppm. MS/MS spectra were acquired in the parallel accumulation-serial fragmentation (PASEF) mode with collision energies of 20 eV and 50 eV. The isolation width was set to increase linearly from *m/z* (20 ± 2) to (1′300 ± 8). N_2_ was used as collision gas. Compass HyStar 6.2 combined with otofControl 6.2 (*Bruker*) were used for data acquisition. DataAnalysis 5.3 (*Bruker*) was used for data processing.

### Purification of phymabactin C8

The bacterial supernatant of *P. phymatum* (500 mL) was desalted on a column packed with 150 mL polymeric resin (Amberlite™ XAD16N, *Sigma Aldrich*). The column was conditioned with 500 mL H_2_O. Then, 500 mL bacterial supernatant was adsorbed on the column, washed with 500 mL H_2_O and eluted with 500 mL MeOH. This eluate was dried *in vacuo* (40 °C, 120 mbar) and reconstituted in 5 mL H_2_O to obtain a crude extract.

The siderophore phymabactin C8 (**7**) was purified by semi-preparative HPLC on a *Shimadzu* system built from two LC-20AP pumps, a DGU-20A3 degasser, a CBM-20A communication bus module, an SPD-20A UV/Vis detector and an FRC-10A fraction collector. Chromatographic separation was performed at room temperature with an XSelect HSS C18 OBD Prep column (100 Å, 5 µm particle size, 10 × 150 mm, *Waters*). The mobile phase consisted of A: H_2_O + 0.1% HCOOH and B: CH_3_CN + 0.1% HCOOH. A constant flow rate of 5 mL/min was applied using the following gradient: (I) 5% B isocratic from 0.0 to 5.0 min; (II) linear increase to 35% B from 5.0 to 25.0 min; (III) linear increase to 100% B from 25.0 to 27.0 min; (IV) 100% B isocratic from 27.0 to 35.0 min. UV absorption was monitored at a wavelength of 220 nm. Five chromatographic runs with identical conditions were performed, injecting 1 mL of crude extract each time. The fractions containing **7** were collected between 20.0 and 21.0 min (see Fig. S2) and combined. After lyophilization, a yellowish powder (1.04 mg) was obtained and redissolved in 200 µL H_2_O + 2% HCOOH.

A second purification step of the combined fractions containing **7** was performed by analytical HPLC on a Vanquish Horizon UHPLC system (*Thermo Fisher*) built from a binary pump H, a split sampler HT, a temperature-controlled column compartment and a diode array detector. Chromatographic separation was performed at 35 °C with an XSelect HSS C18 column (100 Å, 5 µm particle size, 4.6 × 150 mm, *Waters*). The mobile phase consisted of A: H_2_O + 0.1% HCOOH and B: CH_3_CN + 0.1% HCOOH. A constant flow rate of 1 mL/min was applied using the following gradient: (I) 5% B isocratic from 0.0 to 0.5 min; (II) linear increase to 30% B from 0.5 to 20.0 min; (III) linear increase to 100% B from 20.0 to 20.1 min; (IV) 100% B isocratic from 20.1 to 25.0 min; (V) linear decrease to 5% B from 25.0 to 25.1 min; (VI) 5% B isocratic from 25.1 to 30.0 min. UV absorption was monitored at wavelengths from 190 to 680 nm. A total of eight chromatographic runs, each with an injection volume of 25 µL, were performed. The Al(III)−**7** complex eluted between 13.2 and 14.0 min, while **7** eluted between 14.4 and 16.5 min (see Fig. S3). Despite the broad peak shape caused by column overloading, Al(III)−**7** and **7** were baseline-separated. The fractions containing **7** were automatically collected using a six-port valve controlled by retention time (RT) and combined. After lyophilization, phymabactin C8 (**7**, 0.53 mg) was obtained as a white powder and stored at − 20 °C until further analysis.

### NMR spectroscopy

Purified phymabactin C8 (**7**, 0.53 mg) was dissolved in 250 µL of H_2_O/D_2_O (9:1) + 1 mM HCl and transferred to a Shigemi 5 mm NMR microtube. NMR data was acquired on a *Bruker* Avance Neo 600 MHz spectrometer equipped with a 5 mm TCI cryoprobe. The following NMR experiments were conducted: ^1^H with water suppression using excitation sculpting, ^13^C with broadband decoupling, distortionless enhancement by polarization transfer (DEPT) 90, DEPT 135, heteronuclear single quantum correlation (HSQC), double quantum filtered correlation spectroscopy (DQF-COSY), total correlation spectroscopy (TOCSY), nuclear Overhauser effect spectroscopy (NOESY), HSQC-TOCSY and heteronuclear multiple bond correlation (HMBC). The ^1^H NMR chemical shift (*δ*_1H_) was referenced to the H_2_O solvent peak and the ^13^C NMR chemical shift (*δ*_13C_) was indirectly referenced using the ^13^C/^1^H frequency ratio according to the IUPAC recommendations (Harris et al. 2008). Mnova 14.1 (*Mestrelab Research*, Santiago de Compostela, Spain) was used for data processing.

### Hydrogen deuterium exchange

Compound **7** was dissolved separately in H_2_O/CH_3_OH (3:2) and D_2_O/CD_3_OD (3:2), both at a concentration of 10 µg/mL, and analyzed on a timsTOF Pro QTOF mass spectrometer (*Bruker*) via continuous flow injection (3 µL/min). The mass spectrometer was operated in the positive ESI mode at 3′000 V capillary voltage and 500 V endplate offset with a N_2_ nebulizer pressure of 0.4 bar and a dry gas flow of 4 L/min at 180 °C. MS/MS spectra were acquired in the multiple reaction monitoring (MRM) mode at a collision energy of 20 eV and an isolation window of 6 *m*/*z*. Mass calibration was performed as described in the UHPLC-TIMS-HRMS method.

### Amino acid analysis

The absolute configuration of the amino acids was determined based on Marfey’s method (Marfey 1984). The siderophore **7** (15 µg) was hydrolyzed under a stream of 6 M HCl + 0.1% phenol (*w*/*v*) at 110 °C for 24 h. Derivatization of the hydrolyzed amino acids was performed using the advanced Marfey’s reagent D-FDLDA according to the procedure described in literature (Ozaki et al. 2022). Derivatized amino acids were analyzed on an ACQUITY UPLC system coupled to a QDa single quadrupole mass detector (*Waters*). Chromatographic separation was performed at 55 °C with a Cortecs C18 column (90 Å, 1.6 µm particle size, 2.1 × 150 mm, *Waters*). The injection volume was 1 µL. The mobile phase consisted of A: H_2_O + 0.1% HCOOH and B: CH_3_CN + 0.1% HCOOH. A constant flow rate of 0.5 mL/min was applied using the following gradient: (I) 1% B isocratic from 0.0 to 1.0 min; (II) linear increase to 85% B from 1.0 to 8.5 min; (III) linear increase to 95% B from 8.5 to 8.6 min; (IV) 95% B isocratic from 8.6 to 9.5 min; (V) linear decrease to 1% B from 9.5 to 9.6 min; (VI) 1% B isocratic from 9.6 to 15.0 min.

## Results and discussion

### Siderophore screening via complexation

Siderophores produced by *P. phymatum* and *P. tuberum*::*orb* were identified using our in-house screening assay (Rehm et al. 2023a). For this purpose, bacterial supernatants were spiked with ferric iron and complexation was monitored by UHPLC-TIMS-HRMS analysis. The presence of iron in an isotope pattern distribution was easy to recognize by means of the ^54^Fe isotope (X − 2), which has a relative abundance of 5.8%. Therefore, a specific shift of the isotope distribution, mass and RT was expected upon complexation of siderophores with iron. Siderophores identified in *Paraburkholderia* strains were detected as [*M* + 2H]^2+^ ions before complexation and [*M* + Fe − H]^2+^ ions after complexation, which led to a mass shift of 26.4557 Da.

**Fig. 2:**
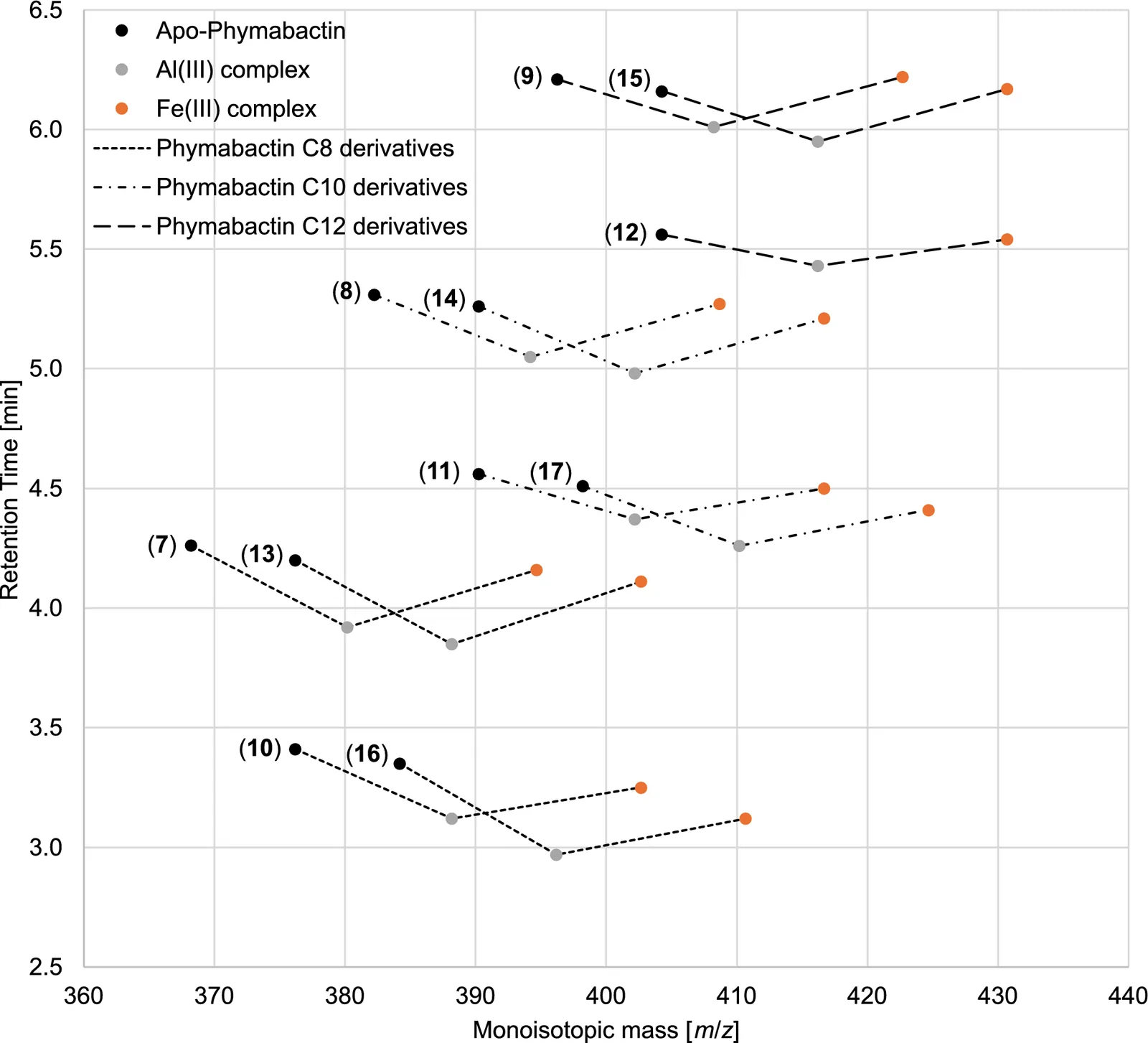
Retention times (RT) of phymabactin derivatives (**7**‒**17**) found in *P. phymatum* supernatant plotted against their monoisotopic masses. Al(III) and Fe(III) were spiked to the bacterial supernatant to check the formation of siderophore-metal complexes

A total of eleven siderophores were identified in *P. phymatum* supernatant, all exhibiting the expected isotope distribution and *m*/*z* shifts (see Fig. 2, S4 and S5). An average RT shift of −0.08 min was observed for iron complexation. The most abundant siderophore was detected at RT 4.26 min with *m*/*z* 368.2163 (C_31_H_60_N_8_O_12_^2+^, 0.9 ppm), which shifted to RT 4.16 min with *m*/*z* 394.6720 (C_31_H_57_N_8_O_12_Fe^2+^, 0.6 ppm) after complexation with iron. Thus, a neutral molecular formula *M* = C_31_H_58_N_8_O_12_ was derived using elemental composition calculations based on HRMS data (Kind and Fiehn 2007). A comprehensive search of both public and in-house databases revealed no known siderophore with the specified molecular formula or MS/MS spectrum. Consequently, we were able to confirm the production of a novel family of siderophores by *P. phymatum*, designated as phymabactins, in accordance with the initial annotation of the phymabactin gene cluster (Esmaeel et al. 2016).

Surprisingly, for each identified phymabactin derivative, an additional feature shifted by 11.9790 Da eluted around 0.3 min earlier in the supernatant of *P. phymatum* (see Fig. S4 and S5). For the most abundant phymabactin derivative, this feature was detected at RT 3.92 min with *m*/*z* 380.1952 (C_31_H_57_N_8_O_12_Al^2+^, 0.5 ppm). The isotope pattern did not show a distinct change compared to the apo-phymabactins, indicating the presence of a monoisotopic element, which was assumed to be aluminum. In fact, complexation experiments with Al(III) confirmed the presence of aluminum-phymabactin complexes ([*M* + Al − H]^2+^) as shown in Fig. 2. Related hydroxamate siderophores were reported to form aluminum-complexes as described for ornibactins (Mathew et al. 2016), acremonpeptides (Luo et al. 2019) and deferoxamine B (Bellotti and Remelli 2021).

As expected, three ornibactin derivatives (**1**‒**3**) were identified in *P. tuberum*::*orb* supernatant. MS/MS spectra of **1**‒**3** matched with reference spectra retrieved from Global Natural Products Social Molecular Networking (Aron et al. 2020). In contrast to the phymabactin complexes, the aluminum and iron complexes of ornibactin were detected with a delayed RT. An overview of all identified phymabactin and ornibactin siderophores is depicted in Table S1.

### Structure elucidation of phymabactin C8

**Fig. 3:**
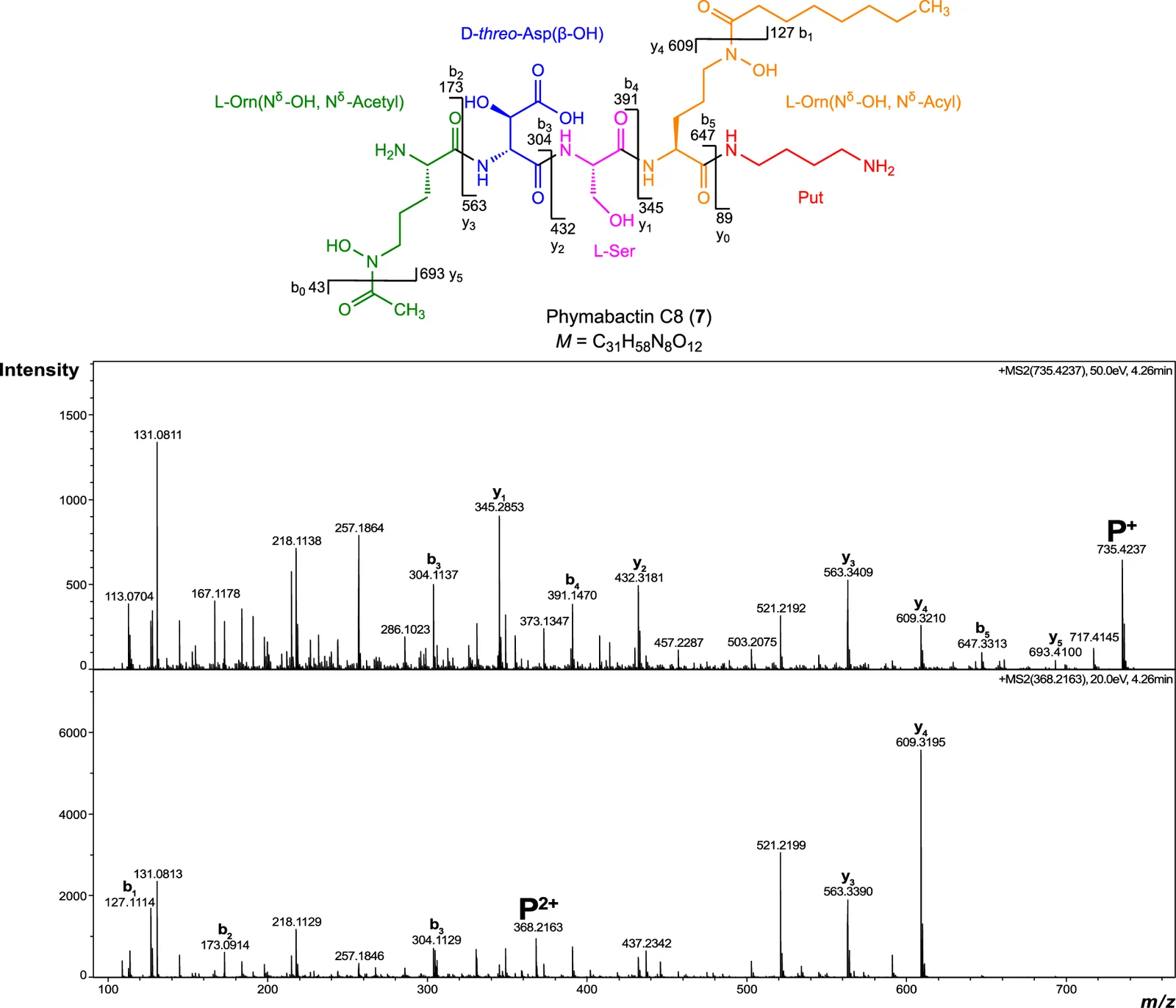
Structural formula of **7** and corresponding MS/MS spectra, labeled with B and Y fragment ions. The single-charged precursor ion at *m/z* 735 (P^+^) and double-charged at *m/z* 368 (P^2+^) were fragmented at collision energies of 50 and 20 eV, respectively

**Fig. 4:**
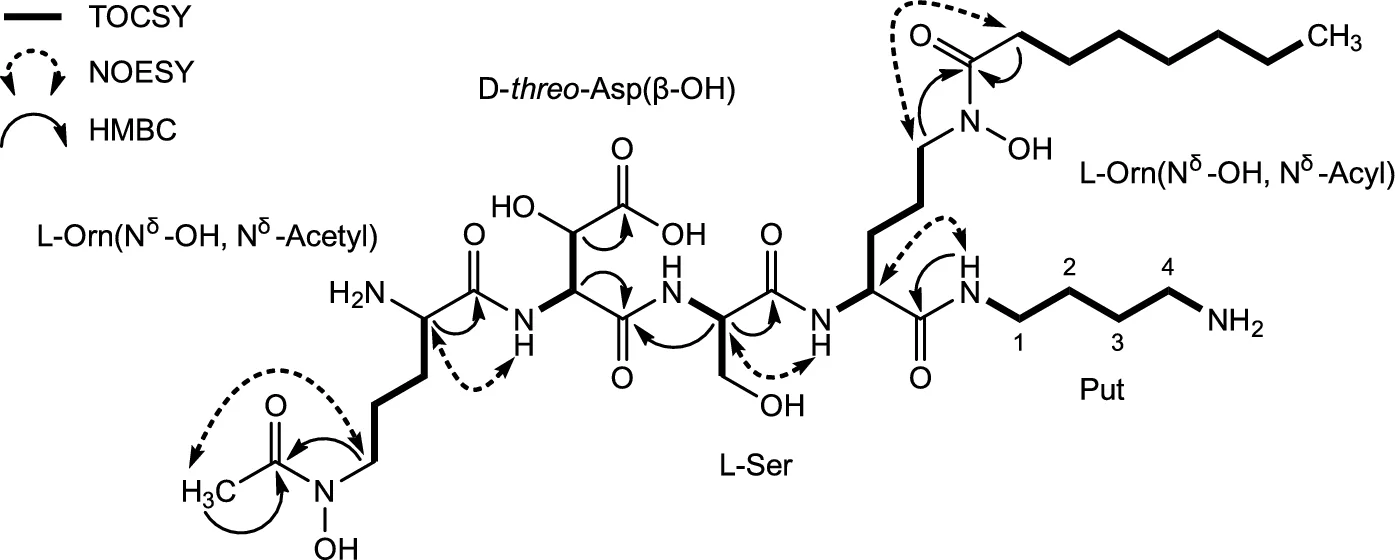
Structure of **7** elucidated by NMR including key TOCSY (bold bonds), NOESY (dashed arrow, H/H) and HMBC (plain arrow, H/C) correlations

The most abundant siderophore produced by *P. phymatum*, phymabactin C8 (**7**), was purified by semi-preparative liquid chromatography as described in the materials and methods section. Its molecular structure was elucidated by means of MS/MS fragmentation (Fig. 3), various NMR experiments (Fig. 4 and Table 1) and amino acid analysis (Table S2).

**Table 1:** ^1^H and ^13^C NMR data of **7** (H_2_O/D_2_O 9:1 + 1 mM HCl, 298 K)

Residue	Position	*δ*_13C_ [ppm]	*δ*_1H_ [ppm]	^1^H Multiplicity
Orn(N^δ^-OH, N^δ^-Acetyl)	NH_2_	–	n.d	–
	C=O	172.9	–	–
	α-CH	55.7	4.15	t, *J* = 6.4 Hz
	β-CH_2_	30.9	1.84	m
	γ-CH_2_	23.7	1.61	m
	δ-CH_2_	49.8	3.55 3.75	m m
Acetyl	CH_3_	22.1	2.14	s
	C=O	176.8	–	–
Asp(β-OH)	NH	–	8.62	d, *J* = 8.6 Hz
	C=O	174.6	–	–
	α-CH	59.5	4.91	m
	β-CH	74.9	4.51	d-like m
	γ-COOH	178.8	n.d	–
Ser	NH	–	8.37	d, *J* = 7.1 Hz
	C=O	174.6	–	–
	α-CH	58.8	4.45	t-like m
	β-CH_2_	63.6	3.85 3.88	dd, *J* = 11.7, 4.9 Hz dd, *J* = 11.7, 5.7 Hz
Orn(N^δ^-OH, N^δ^-Acyl)	NH	–	8.25	d, *J* = 6.8 Hz
	C=O	176.3	–	–
	α-CH	56.9	4.22	m
	β-CH_2_	30.6	1.67 1.75	m
	γ-CH_2_	25.2	1.61 1.69	m
	δ-CH_2_	50.0	3.60 3.64	m
Acyl	C=O	179.3	–	–
	α-CH_2_	34.4	2.46	t-like m
	β-CH_2_	27.2	1.53	m
	γ-CH_2_	31.1	1.26	m
	δ-CH_2_	30.8	1.26	m
	ε-CH_2_	33.6	1.22	m
	ζ-CH_2_	24.6	1.23	m
	η-CH_3_	16.1	0.81	t, *J* = 7.2 Hz
Put	NH	–	7.94	t, *J* = 6.1 Hz
	1-CH_2_	41.1	3.20	m
	2-CH_2_	28.1	1.54	m
	3-CH_2_	26.9	1.61	m
	4-CH_2_	42.1	2.96	broad t, *J* = 7.5 Hz
	NH_2_	–	7.50	broad

Different MS techniques including elemental composition calculation, fragmentation by collision-induced dissociation (CID) and hydrogen deuterium exchange were applied. Elemental composition calculations of the [*M* + 2H]^2+^ ion at *m*/*z* 368.2163 (0.8 ppm) and the less intense [*M* + H]^+^ ion at* m*/*z* 735.4237 (− 1.4 ppm) both led to the neutral molecular formula of *M* = C_31_H_58_N_8_O_12_. With this formula, a ring and double bond equivalent (RDB) of seven was calculated. The collision energies in MS/MS experiments were optimized to obtain the greatest number of fragment ions while retaining the precursor ions. An optimal collision energy of 20 eV was determined for the double-charged precursor ion, while 50 eV were required to fragment the single-charged precursor ion (Fig. 3). Breakage of amide bonds in **7** led to characteristic B and Y fragments according to MS/MS fragmentation rules in peptides (Paizs and Suhai 2005). Such a characteristic fragment was observed at *m*/*z* 609.3195 (C_23_H_45_N_8_O_11_^+^, − 1.1 ppm) with a counterpart at *m*/*z* 127.1114 (C_8_H_15_O^+^, − 2.4 ppm) revealing an acyl chain with a length of eight carbons. Additional pairs of B and Y fragments were detected at *m*/*z* 173.0914 (C_7_H_13_N_2_O_3_^+^, − 4.0 ppm) and 563.3390 (C_24_H_47_N_6_O_9_^+^, − 1.6 ppm), *m*/*z* 304.1137 (C_11_H_18_N_3_O_7_^+^, − 0.7 ppm) and 432.3181 (C_20_H_42_N_5_O_5_^+^, 0.2 ppm), and *m*/*z* 391.1470 (C_14_H_23_N_4_O_9_^+^, 2.6 ppm) and 345.2853 (C_17_H_37_N_4_O_3_^+^, − 2.0 ppm). These characteristic fragments confirmed the amino acid sequence [Orn-Asp(β-OH)-Ser-Orn-Put] consistent with ornibactin (**1**‒**3**) and malleobactin (**4**‒**6**) siderophores as shown in Fig. 1 (Franke et al. 2013; Stephan et al. 1993). Hydrogen deuterium exchange experiments of **7** revealed the presence of 13 exchangeable protons (see Fig. S6). By adding one acyl (C_8_H_15_O), one acetyl (C_2_H_3_O), and two hydroxyl (OH) moieties to the sequence [Orn-Asp(β-OH)-Ser-Orn-Put], the number of exchangeable protons and the molecular formula of **7** are completed. However, these moieties and their exact position still needed to be confirmed by NMR spectroscopy.

In the next step, 1D and 2D NMR spectra of **7** were evaluated to determine its explicit structure. The compound was dissolved in H_2_O/D_2_O (9:1) adjusted to a pH of 3, allowing us to detect signals of exchangeable amide protons. All NMR spectra employed for structure elucidation are provided in the supplementary (Fig. S7 – S18). The chemical shifts of protons (*δ*_1H_) and carbon atoms (*δ*_13C_) are listed in Table 1. The ^13^C NMR spectrum enabled the detection of 31 carbon atoms, in accordance with the proposed molecular formula. Protons were assigned to linked carbon atoms using the HSQC spectrum and ^13^C multiplicities were deduced from DEPT90 and DEPT135 spectra. Seven carbonyl resonances were detected between *δ*_13C_ 172.9 and 179.3 ppm, which account for all RDB equivalents. Four α-CH groups were detected with *δ*_1H_ between 4.15 and 4.91 ppm and *δ*_13C_ between 55.7 and 59.5 ppm confirming the presence of four amino acids. The α-proton of Asp(β-OH) at *δ*_1H_ 4.91 ppm exhibited DQF-COSY coupling with an amide backbone proton at *δ*_1H_ 8.62 ppm. When switching to the TOCSY spectrum, the α-proton and the β-proton (*δ*_1H_ 4.51 ppm) of Asp(β-OH) showed coupling with the same amide proton (see Fig. S14). The spin systems of Ser and Orn(N^δ^-OH, N^δ^-Acyl) were identified in the same manner, starting from their α-protons at *δ*_1H_ 4.45 and 4.21 ppm, respectively. Amide backbone protons were detected at *δ*_1H_ 8.37 ppm for Ser and at *δ*_1H_ 8.25 ppm for Orn(N^δ^-OH, N^δ^-Acyl). The α-proton of Orn(N^δ^-OH, N^δ^-Acetyl) at *δ*_1H_ 4.15 ppm did not show any backbone coupling due to its N-terminal position. A triplet at *δ*_1H_ 7.94 ppm, which showed DQF-COSY coupling with a CH_2_ group at *δ*_1H_ 3.20 ppm, was assigned to the amide proton of the Put residue. A total of four CH_2_ groups were assigned to the spin system of Put and their chemical shifts agreed with NMR data of malleobactins (Franke et al. 2013). Overall, six spin systems were identified using TOCSY correlations, all depicted in Fig. 4.

The assignment of carbonyl resonances to their neighboring atoms was achieved through *J*_H,C_ coupling visible in the HMBC spectrum. For example, the α-CH_2_ group of the acyl residue at *δ*_1H_ 2.46 ppm and the δ-CH_2_ group of Orn at *δ*_1H_ 3.60 and 3.64 ppm both correlated with the carbonyl group at *δ*_13C_ 179.3 ppm. On the other hand, the methyl group at *δ*_1H_ 2.14 ppm and the δ-CH_2_ group of Orn at *δ*_1H_ 3.55 and 3.75 ppm both showed HMBC correlations with the carbonyl resonance at *δ*_13C_ 176.8 ppm. This corroborated the presence of an Orn(N^δ^-OH, N^δ^-Acetyl) moiety in N-terminal position. The NOESY spectrum correlates protons that are spatially close to each other and was therefore used to connect neighboring spin systems. Hence, the Orn residue with an α-CH proton at *δ*_1H_ 4.22 ppm exhibited NOESY correlation with the amide proton of Put. Furthermore, the δ-CH_2_ protons of the same Orn residue showed a NOESY signal with the α-CH_2_ protons of the acyl residue. These correlations demonstrated the presence of an Orn(N^δ^-OH, N^δ^-Acyl) moiety located between the Ser and Put residues. All key HMBC and NOESY correlations are depicted in Fig. 4, and the spectra are provided in Fig. S13, S17 and S18.

To complete the structure elucidation of **7**, the absolute configurations of its four amino acid moieties were determined by Marfey’s method (Marfey 1984). After hydrolysis of the amide bonds, the free amino groups were derivatized with D-FDLDA, which enabled chromatographic separation of the D- and L-amino acids (Kuranaga et al. 2020). Obtained RT (see Table S2) were compared to enantiopure amino acid standards undergoing the same derivatization procedure. Due to the presence of two amino groups in Orn, two chromatographic peaks were detected, both in agreement with the L-Orn standard. Furthermore, the presence of D-*threo*-Asp(β-OH) and L-Ser was confirmed using enantiopure reference standards. These results agree with absolute configurations reported for the same amino acids present in ornibactins (Stephan et al. 1993) and malleobactins (Franke et al. 2015).

### Structure elucidation of phymabactin derivatives

Siderophore screening of *P. phymatum* supernatant revealed ten additional phymabactin derivatives (**8**‒**17**) that were less abundant than **7**. With the elucidated structure of **7** serving as a starting point, it was possible to elucidate these metabolites by elemental composition and CID fragmentation analysis without prior purification. MS/MS spectra of **7**‒**17** acquired in the PASEF mode together with their structural formulas are provided in the supplementary (Fig. S19 – S23).

The following three generic molecular formulas were obtained for phymabactins by elemental composition calculations: C_31_H_58_N_8_O_x_, C_33_H_62_N_8_O_x_ and C_35_H_66_N_8_O_x_ (x = 12, 13 or 14). All these molecular formulas correspond to an RDB equivalent of seven, hence a constant number of seven carbonyl double bonds across all phymabactins was expected. Elongated acyl chains were identified as the cause of the C_2_H_4_ differences between the molecular formulas. Characteristic B fragments confirming an elongated acyl chain were detected at *m*/*z* 155.1433 (C_10_H_19_O^+^, 1.9 ppm) for phymabactin C10 (**8**) and at *m*/*z* 183.1732 (C_12_H_23_O^+^, − 6.0 ppm) for phymabactin C12 (**9**). Due to backbones identical to **7**, the corresponding Y fragment remained at *m*/*z* 609 (C_23_H_45_N_8_O_11_^+^) in **8** and **9**. The order of elution of **7**, **8** and **9** at RT 4.26, 5.31 and 6.21 min, respectively, was consistent with the increasing lipophilicity of the elongated acyl chains. MS/MS peak lists and chemical identifiers of **7**‒**9** are provided in Table S3 and S4.

Several oxidation products of **7**‒**9** have been found which contain one or two additional oxygen atoms. A first hydroxylation site was observed at the acyl residue and confirmed by the preserved Y fragment ion at *m*/*z* 609.3199 (C_23_H_45_N_8_O_11_^+^, − 0.5 ppm). Corresponding B fragments underwent water loss and were consequently detected at *m*/*z* 125.0959 (C_8_H_13_O^+^, − 1.6 ppm), 153.1275 (C_10_H_17_O^+^, 0.7 ppm) and 181.1574 (C_12_H_21_O^+^, − 7.2 ppm) depending on the acyl chain length. These phymabactin derivatives **10**‒**12** were named according to their C8, C10 or C12 acyl chain, followed by the suffix Ac(OH). An increase in polarity of **10**‒**12** was demonstrated by an average RT shift of − 0.75 min relative to **7**‒**9**. Hydroxylation of the acyl chain was assumed to take place at β-position, in agreement with the β-oxidation spiral during fatty acid degradation (Schulz 1991). Siderophores produced by marine and terrestrial bacteria exhibit β-hydroxylated acyl chains, as described for various amphibactins, aquachelin I, loihichelin B, cupriachelin and ornibactins (Kem and Butler 2015; Kreutzer et al. 2012; Stephan et al. 1993).

Another hydroxylation site was located at the Put residue and confirmed by the fragment ion at *m*/*z* 105.1020 (C_4_H_13_N_2_O^+^, − 1.9 ppm). These phymabactin derivatives **13**‒**15** were named according to their C8, C10 or C12 acyl chain, followed by the suffix Put(OH). In contrast to the hydroxylation of the acyl chain, the hydroxylation of the Put residue resulted only in a minor RT shift of − 0.05 min relative to **7**‒**9**. Due to this small change in polarity, we suspect the oxidation of the amide into a hydroxamic acid group, rather than a hydroxylation of the hydrocarbon chain within Put. Finally, two double hydroxylated phymabactin derivatives **16**‒**17** featuring C8 and C10 acyl chains were detected. Both exhibited a Y fragment ion at *m*/*z* 625.3153 (C_23_H_45_N_8_O_12_^+^, 0.3 ppm), which confirms the loss of a hydroxylated acyl chain and is characteristic for phymabactins with a hydroxylated Put residue.

### Ion mobility patterns during metal complex formation

The coupling of ion mobility spectrometry with UHPLC-HRMS offers an additional separation dimension based on the size, shape and charge of analyte ions (Gabelica and Marklund 2018). The generated CCS values are small areas, typically indicated in Å^2^ units, and can be used to differentiate isobaric analytes (Celma et al. 2020). In the case of metallophores, CCS values relate to their shape and provide additional criteria for distinguishing between the apo and bound states (Gomes et al. 2024; Rehm et al. 2023b). Therefore, CCS values of ornibactins **1**‒**3**, phymabactins **7**‒**17** and their corresponding metal complexes were determined in triplicate by UHPLC-TIMS-HRMS analysis. The double-charged molecular ions [*M* + 2H]^2+^, [*M* + Fe – H]^2+^ and [*M* + Al – H]^2+^ were used to extract mobilograms and derive CCS values.

**Fig. 5:**
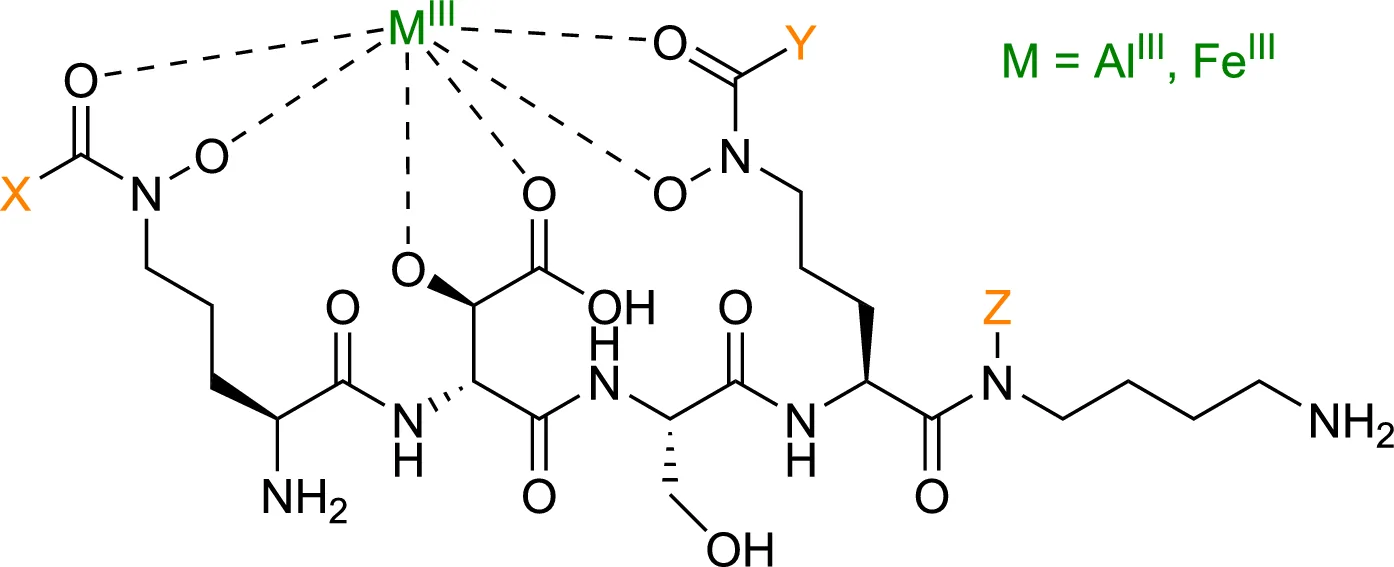
Suggested metal-binding mode of ornibactins **1**‒**3** and phymabactins **7**‒**17** according to literature (Butt and Thomas 2017; Stephan et al. 1993). Substituents X, Y and Z are indicated in Table 2

Ornibactins and phymabactins can both be classified as mixed-type hydroxamate and carboxylate bacterial siderophores (Khan et al. 2018). As shown in Fig. 5, one bidentate hydroxy-carboxylate and two bidentate hydroxamate ligands are involved in hexadentate metal complexation (Butt and Thomas 2017). Although the complex formation constants of phymabactins and ornibactins have not been determined quantitatively, we assume that they have similar metal affinities. However, the molecular structures of the two families of siderophores differ (see Table 2), resulting in altered hydrophobicity. Ornibactins contain a β-hydroxylated C4, C6 or C8 N^δ^-acyl chain at the N-terminal Orn residue and are N^δ^-formylated at the C-terminal Orn residue, whereas phymabactins are N^δ^-acetylated at the N-terminal Orn residue and incorporate a C8, C10 or C12 N^δ^-acyl chain at the C-terminal Orn residue. This variety of substituents (see Table 2) led to different three-dimensional shapes, as witnessed by distinctive CCS values of ornibactins, phymabactins, and corresponding metal complexes.

**Table 2:** Overview of ornibactin and phymabactin siderophores analyzed by UHPLC-TIMS-HRMS. Substituents X, Y and Z refer to the structure in Fig. 5

Compound	X	Y	Z
Ornibactin C4 (**1**)	C_3_H_7_O	H	H
Ornibactin C6 (**2**)	C_5_H_11_O	H	H
Ornibactin C8 (**3**)	C_7_H_15_O	H	H
Phymabactin C8 (**7**)	CH_3_	C_7_H_15_	H
Phymabactin C10 (**8**)	CH_3_	C_9_H_19_	H
Phymabactin C12 (**9**)	CH_3_	C_11_H_23_	H
Phymabactin C8 Ac(OH) (**10**)	CH_3_	C_7_H_15_O	H
Phymabactin C10 Ac(OH) (**11**)	CH_3_	C_9_H_19_O	H
Phymabactin C12 Ac(OH) (**12**)	CH_3_	C_11_H_23_O	H
Phymabactin C8 Put(OH) (**13**)	CH_3_	C_7_H_15_	OH
Phymabactin C10 Put(OH) (**14**)	CH_3_	C_9_H_19_	OH
Phymabactin C12 Put(OH) (**15**)	CH_3_	C_11_H_23_	OH
Phymabactin C8 OH-OH (**16**)	CH_3_	C_7_H_15_O	OH
Phymabactin C10 OH-OH (**17**)	CH_3_	C_9_H_19_O	OH

**Fig. 6:**
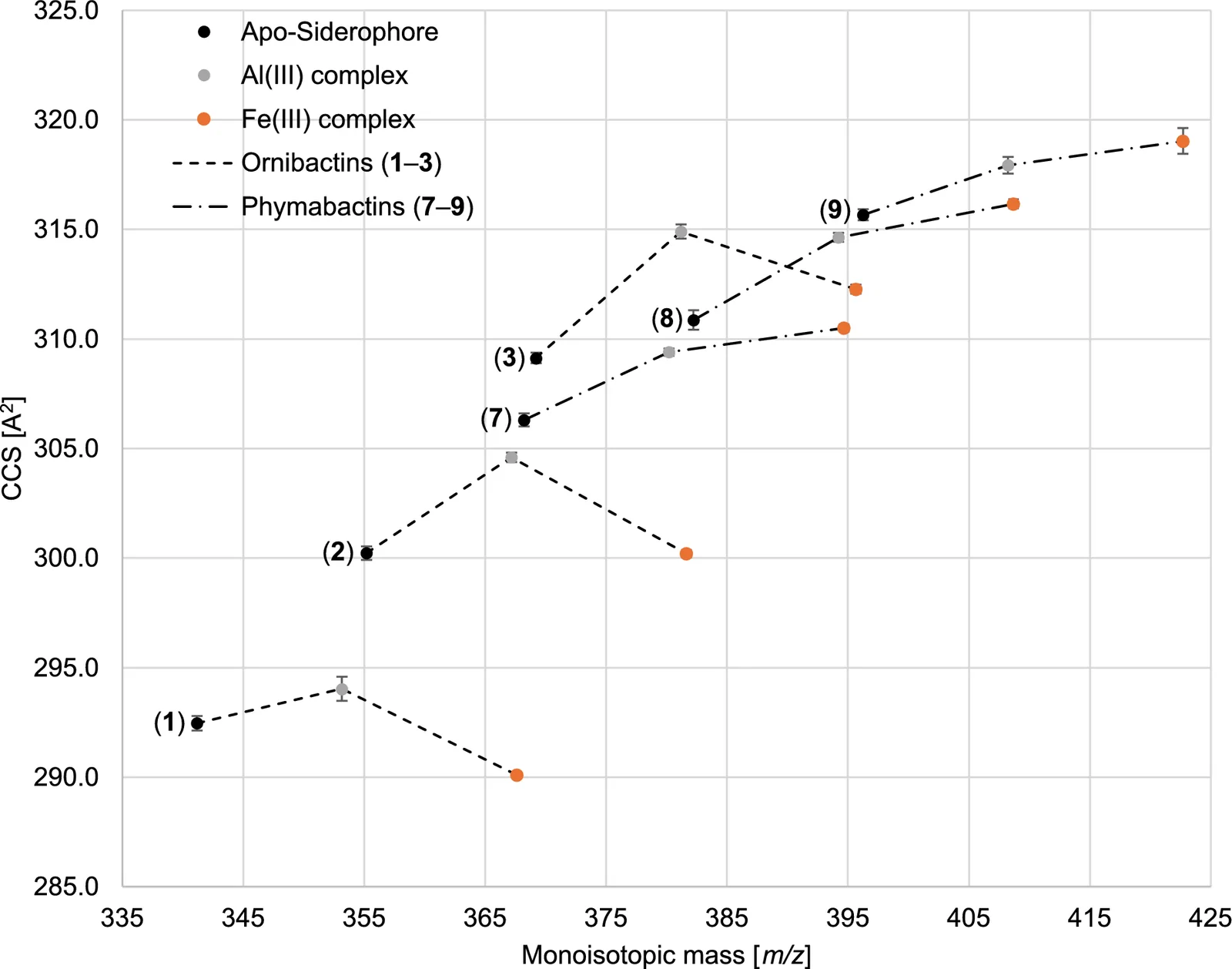
CCS values of the ornibactins **1**‒**3** and phymabactins **7**‒**9** plotted against their monoisotopic masses. CCS values were derived from double-charged molecular ions of apo-siderophores, ferric complexes and aluminum complexes. Error bars represent standard deviations of CCS values

CCS values of ornibactins (**1**‒**3**) and phymabactins (**7**‒**9**) increased by an average of 3.9 and 3.0 Å^2^, respectively, following aluminum complexation (see Fig. 6). Furthermore, CCS values of ferric ornibactins decreased by an average of 3.7 Å^2^ compared to aluminum-ornibactin complexes. Contrarily, the CCS values of ferric phymabactins slightly increased by an average of 1.2 Å^2^ compared to aluminum-phymabactin complexes. Therefore, the ion mobility patterns of ornibactins and phymabactins were clearly distinguishable, as shown in Fig. 6.

**Fig. 7:**
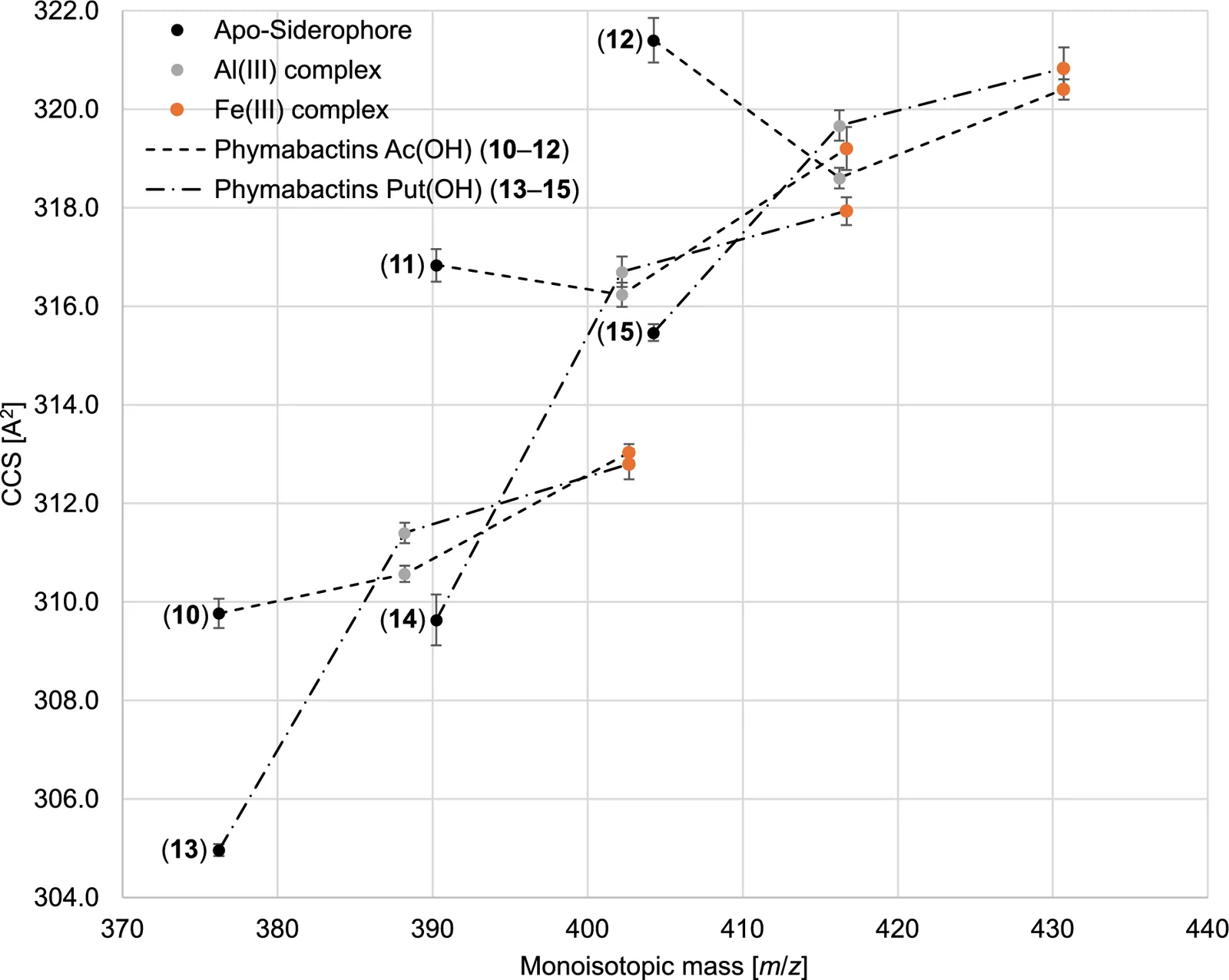
CCS values of the hydroxylated phymabactins **10**‒**15** plotted against their monoisotopic masses. CCS values were derived from double-charged molecular ions of apo-siderophores, ferric complexes and aluminum complexes. Error bars represent standard deviations of CCS values

Hydroxylation of either the acyl chain or the Put residue resulted in three pairs of isobaric phymabactins, which cannot be differentiated by mass spectrometric detection without prior chromatographic separation. In such cases, ion mobility data can provide additional confidence for unambiguous identification of isobaric compounds (Ageitos et al. 2025; Celma et al. 2020). In fact, the CCS values of the phymabactins Ac(OH) **10**‒**12** were on average 6.0 Å^2^ larger than those of the corresponding isobaric phymabactins Put(OH) **13**‒**15** (see Fig. 7). Therefore, hydroxylation of the acyl chain resulted in a less compact form and is likely exposed at the surface of the apo-siderophore. In contrast, the Put residue undergoes hydroxylation closer to the amide backbone resulting in CCS values similar to those of the non-hydroxylated phymabactins **7**‒**9**. The differences between the CCS values of isobaric phymabactins diminished after complexation with aluminum or iron (see Fig. 7), suggesting that the additional hydroxyl groups on the acyl chain or Put residue are both involved in metal complexation.

## Conclusion

We here report for the first time the absolute structure of phymabactin elucidated by MS/MS, NMR and amino acid analysis. A total of eleven phymabactin derivatives were proposed, which all displayed characteristic shifts in polarity (RT), mass (*m*/*z*) and ion mobility (CCS) upon complexation with aluminum and iron. To the best of our knowledge, no ion mobility data of siderophores produced by *Burkholderiaceae* have been published, thus the presented CCS values of phymabactins, ornibactins and their metal complexes are a novelty. These CCS values can facilitate the identification of metallophores in complex bacterial extracts, as exemplified with the ion mobility separation of isobaric phymabactins. Furthermore, ion mobility patterns of phymabactins allow us to assume that the hydroxylated acyl and Put side chains are involved in the metal complexation. In the future, phymabactins could be obtained selectively through solid-phase peptide synthesis, followed by a quantitative determination of their complex formation constants. Further studies may enable their application as chelation therapy, crop protection or bioremediation agents.

## Supplementary Information

Below is the link to the electronic supplementary material.

Supplementary file1 (PDF 5313 KB)

## Data Availability

All data supporting the findings of this study are available within the paper and its Supplementary Information.
