# Supplementary material for: Siderophore-metal complexes in Paraburkholderia phymatum: structure elucidation of phymabactin
Source: Biometals. 2026 May 4;39(5):1681–97. doi: 10.1007/s10534-026-00823-9 (PMC13669132; doi:10.1007/s10534-026-00823-9)
Supplement: Supplementary file 1 [file 10534_2026_823_MOESM1_ESM.pdf]

Supplementary

Siderophore-metal complexes in *Paraburkholderia phymatum*: structure elucidation of phymabactin

Luca Bürgi[1], Daphné Golaz[2], Gabriella Pessi[2] and Laurent Bigler[1*]

[1]Department of Chemistry, University of Zurich, Zurich, Switzerland

[2]Department of Plant and Microbial Biology, University of Zurich, Zurich, Switzerland

***Correspondence:**

Laurent Bigler, laurent.bigler@chem.uzh.ch

Table S1 Overview of phymabactin and ornibactin derivatives investigated in this study. Retention times (RT), monoisotopic masses, and collision cross sections (CCS) with standard deviations (SD) are indicated for each compound as apo-siderophore, aluminum complex and ferric complex.

		Apo-Siderophore $[M + 2H]^{2+}$			Aluminum Complex $[M + Al - H]^{2+}$			Ferric Complex $[M + Fe - H]^{2+}$		
**Name**	**Sum Formula *M***	**RT [min]**	**$m/z_{calculated}$**	**CCS ± SD [Å^2]**	**RT [min]**	**$m/z_{calculated}$**	**CCS ± SD [Å^2]**	**RT [min]**	**$m/z_{calculated}$**	**CCS ± SD [Å^2]**
Phymabactin C8 (**7**)	C31H58N8O12	4.26	368.21599	306.3 ± 0.3	3.92	380.19502	309.4 ± 0.2	4.16	394.67175	310.5 ± 0.2
Phymabactin C10 (**8**)	C33H62N8O12	5.31	382.23164	310.9 ± 0.4	5.05	394.21067	314.6 ± 0.2	5.27	408.68740	316.2 ± 0.2
Phymabactin C12 (**9**)	C35H66N8O12	6.21	396.24729	315.7 ± 0.2	6.01	408.22632	317.9 ± 0.4	6.22	422.70306	319.0 ± 0.6
Phymabactin C8 Ac(OH) (**10**)	C31H58N8O13	3.41	376.21344	309.8 ± 0.1	3.12	388.19248	310.6 ± 0.2	3.25	402.66921	313.0 ± 0.3
Phymabactin C10 Ac(OH) (**11**)	C33H62N8O13	4.56	390.22909	316.8 ± 0.2	4.37	402.20813	316.2 ± 0.3	4.50	416.68486	319.2 ± 0.4
Phymabactin C12 Ac(OH) (**12**)	C35H66N8O13	5.56	404.24474	321.4 ± 0.2	5.43	416.22378	318.6 ± 0.1	5.54	430.70051	320.4 ± 0.1
Phymabactin C8 Put(OH) (**13**)	C31H58N8O13	4.20	376.21344	305.0 ± 0.3	3.85	388.19248	311.4 ± 0.2	4.11	402.66921	312.8 ± 0.4
Phymabactin C10 Put(OH) (**14**)	C33H62N8O13	5.26	390.22909	309.6 ± 0.5	4.98	402.20813	316.7 ± 0.2	5.21	416.68486	317.9 ± 0.3
Phymabactin C12 Put(OH) (**15**)	C35H66N8O13	6.16	404.24474	315.5 ± 0.2	5.95	416.22378	319.7 ± 0.2	6.17	430.70051	320.8 ± 0.4
Phymabactin C8 OH-OH (**16**)	C31H58N8O14	3.35	384.21090	310.9 ± 0.5	2.97	396.18993	311.6 ± 0.3	3.12	410.66667	310.5 ± 0.3
Phymabactin C10 OH-OH (**17**)	C33H62N8O14	4.51	398.22655	317.5 ± 0.3	4.26	410.20558	316.5 ± 0.2	4.41	424.68232	315.8 ± 0.3
Ornibactin C4 (**1**)	C26H48N8O13	0.76	341.17432	292.5 ± 0.3	0.76	353.15335	294.0 ± 0.5	0.80	367.63005	290.1 ± 0.1
Ornibactin C6 (**2**)	C28H52N8O13	1.99	355.18997	300.2 ± 0.3	2.00	367.16900	304.6 ± 0.2	2.30	381.64570	300.2 ± 0.2
Ornibactin C8 (**3**)	C30H56N8O13	3.26	369.20562	309.1 ± 0.2	3.34	381.18465	314.9 ± 0.3	3.58	395.66135	312.3 ± 0.2

Table S2 RT of amino acids in **7** and reference amino acids determined by Marfey's method.

Amino acid	RT of phymabactin C8 [min]	RT of references [min]
L-Orn	5.01 and 5.18	5.02 and 5.19
D-Orn	Not detected	4.69 and 5.23
L-*threo*-Asp(β-OH)	Not detected	5.29
D-*threo*-Asp(β-OH)	5.49	5.47
L-Ser	5.73	5.73
D-Ser	Not detected	5.41

Table S3 MS/MS peak list of **7–9**. Double-charged $[M + 2H]^{2+}$ precursor ions were fragmented at a stepped collision energy of 20 and 50 eV.

Phymabactin C8 (7)			Phymabactin C10 (8)			Phymabactin C12 (9)		
m/z	Absolute Intensity	Relative Intensity [%]	*m/z*	Absolute Intensity	Relative Intensity [%]	*m/z*	Absolute Intensity	Relative Intensity [%]
39.0231	593	3.2	39.0231	474	3.1	41.0393	151	13.1
41.0388	792	4.3	41.0388	1132	7.5	43.0547	253	21.8
43.0185	309	1.7	43.0187	356	2.3	57.0700	641	55.4
43.0543	353	1.9	43.0547	1169	7.7	60.0449	118	10.2
44.0494	427	2.3	55.0539	657	4.3	67.0554	131	11.3
55.0545	376	2.0	57.0702	1988	13.1	70.0650	244	21.1
57.0699	10653	57.8	60.0444	982	6.5	71.0863	170	14.7
60.0443	1571	8.5	67.0544	321	2.1	72.0809	688	59.5
67.0541	548	3.0	68.0497	1242	8.2	72.1013	111	9.6
68.0496	1633	8.9	69.0336	467	3.1	81.0700	114	9.8
69.0333	715	3.9	69.0578	308	2.0	83.0856	179	15.4
70.0281	363	2.0	70.0650	4205	27.7	85.1006	101	8.7
70.0648	6213	33.7	71.0859	1978	13	86.0602	1157	100.0
72.0444	1121	6.1	72.0447	796	5.2	109.1005	163	14.1
72.0807	7707	41.9	72.0812	8979	59.1	113.0718	106	9.2
73.0837	425	2.3	80.0498	341	2.2	114.0555	104	9.0
82.0644	513	2.8	81.0701	1387	9.1	131.0798	176	15.2
86.0600	18415	100.0	85.1007	1034	6.8	173.0930	172	14.9
87.0629	384	2.1	86.0602	15193	100.0	183.1732	101	8.7
89.1074	1302	7.1	87.0630	367	2.4	305.1611	238	20.6
92.0495	488	2.6	89.1070	901	5.9	521.2212	207	17.9
109.1008	743	4.0	95.0604	377	2.5	609.3198	429	37.1
113.0711	386	2.1	95.0857	1282	8.4	610.3218	104	9.0
114.0545	3392	18.4	104.0355	341	2.2	619.4006	106	9.2
127.0861	1782	9.7	113.0716	708	4.7			
127.1122	1764	9.6	114.0554	3634	23.9			
128.0704	745	4.0	127.0863	1367	9.0			
131.0812	3175	17.2	128.0708	430	2.8			
145.0978	944	5.1	131.0818	2290	15.1			
146.0448	421	2.3	145.0959	509	3.4			
173.0915	418	2.3	146.0439	525	3.5			
215.2135	457	2.5	155.1433	1342	8.8			
218.1134	1024	5.6	173.0919	425	2.8			
267.6465	355	1.9	218.1140	649	4.3			
286.1029	342	1.9	243.2433	404	2.7			
304.1136	528	2.9	286.1026	326	2.1			
305.1643	495	2.7	304.1146	468	3.1			
330.7003	451	2.4	305.1653	429	2.8			
331.1233	499	2.7	344.7152	582	3.8			
349.1356	502	2.7	349.1327	300	2.0			
368.2157	542	2.9	373.2279	358	2.4			
368.7179	354	1.9	382.2326	685	4.5			
391.1442	960	5.2	437.2380	389	2.6			
437.2367	335	1.8	460.3490	357	2.3			
446.1890	372	2.0	521.2202	2118	13.9			
521.2200	2022	11.0	522.2213	485	3.2			
522.2245	366	2.0	591.3709	1527	10.0			
563.3412	1154	6.3	592.3749	380	2.5			
609.3215	3367	18.3	609.3202	2703	17.8			
610.3225	891	4.8	610.3230	788	5.2			

Table S4 Chemical identifiers of **7–9** including SMILES, InChI and InChI key. Identifiers generated with ChemDraw 23.1.

**Name**	**SMILES**	**InChI**	**InChI key**
Phymabactin C8 (**7**)	N[C@@H](CCCN(O)C(C)=O)C(N[C@@H](C(N[C@H](C(N[C@H](C(NCCCCN)=O)CCCN(O)C(CCCCCCC)=O)=O)CO)=O)[C@@H](O)C(O)=O)=O	InChI=1S/C31H58N8O12/c1-3-4-5-6-7-14-24(42)39(51)18-11-13-22(28(45)34-16-9-8-15-32)35-29(46)23(19-40)36-30(47)25(26(43)31(48)49)37-27(44)21(33)12-10-17-38(50)20(2)41/h21-23,25-26,40,43,50-51H,3-19,32-33H2,1-2H3,(H,34,45)(H,35,46)(H,36,47)(H,37,44)(H,48,49)/t21-,22-,23-,25+,26+/m0/s1	OAANPMCDQNLPTN-AZQMGYJQSA-N
Phymabactin C10 (**8**)	N[C@@H](CCCN(O)C(C)=O)C(N[C@@H](C(N[C@H](C(N[C@H](C(NCCCCN)=O)CCCN(O)C(CCCCCCCCC)=O)=O)CO)=O)[C@@H](O)C(O)=O)=O	InChI=1S/C33H62N8O12/c1-3-4-5-6-7-8-9-16-26(44)41(53)20-13-15-24(30(47)36-18-11-10-17-34)37-31(48)25(21-42)38-32(49)27(28(45)33(50)51)39-29(46)23(35)14-12-19-40(52)22(2)43/h23-25,27-28,42,45,52-53H,3-21,34-35H2,1-2H3,(H,36,47)(H,37,48)(H,38,49)(H,39,46)(H,50,51)/t23-,24-,25-,27+,28+/m0/s1	HEXCISQXPBXEMD-BJPVBTOCSA-N
Phymabactin C12 (**9**)	N[C@@H](CCCN(O)C(C)=O)C(N[C@@H](C(N[C@H](C(N[C@H](C(NCCCCN)=O)CCCN(O)C(CCCCCCCCCCC)=O)=O)CO)=O)[C@@H](O)C(O)=O)=O	InChI=1S/C35H66N8O12/c1-3-4-5-6-7-8-9-10-11-18-28(46)43(55)22-15-17-26(32(49)38-20-13-12-19-36)39-33(50)27(23-44)40-34(51)29(30(47)35(52)53)41-31(48)25(37)16-14-21-42(54)24(2)45/h25-27,29-30,44,47,54-55H,3-23,36-37H2,1-2H3,(H,38,49)(H,39,50)(H,40,51)(H,41,48)(H,52,53)/t25-,26-,27-,29+,30+/m0/s1	ARQYCLCJYXRRFS-DDAAUBIUSA-N

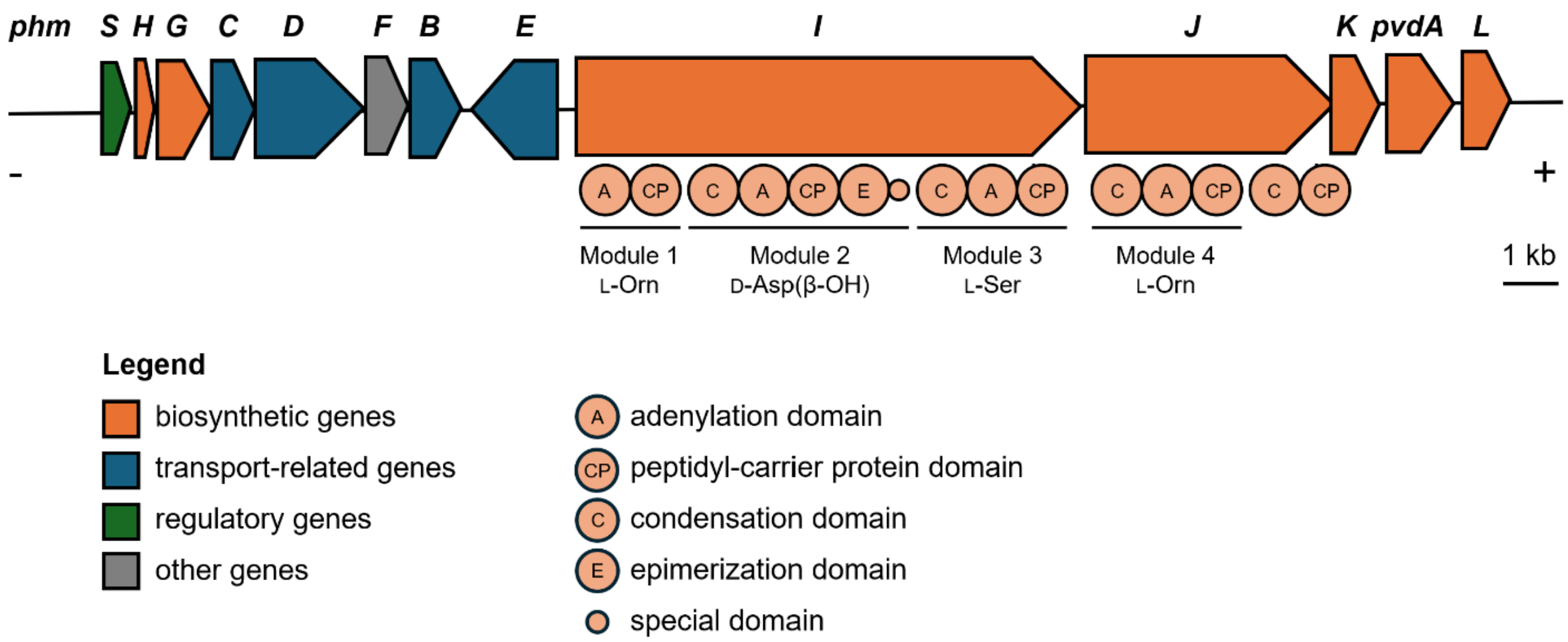

Fig. S1 Biosynthetic gene cluster for phymabactin production, including gene names and color-coded function. Four modules within the non-ribosomal peptide synthetase genes *phmI* and *phmJ* are responsible for the amino acid sequence of phymabactins (**7**–**17**).

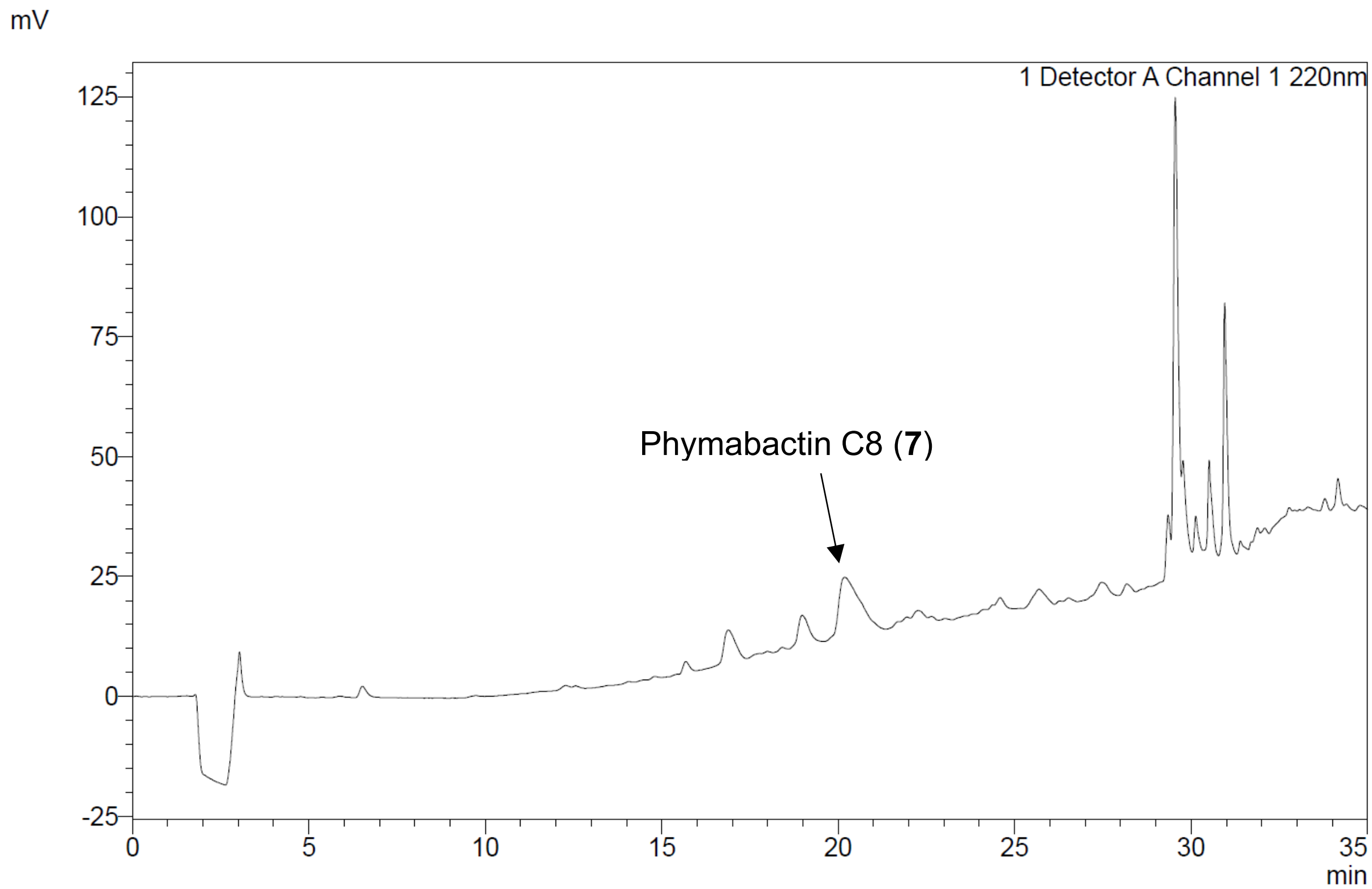

Fig. S2 Purification of **7** by semi-preparative HPLC. The UV chromatogram with a detection wavelength of 220 nm is shown.

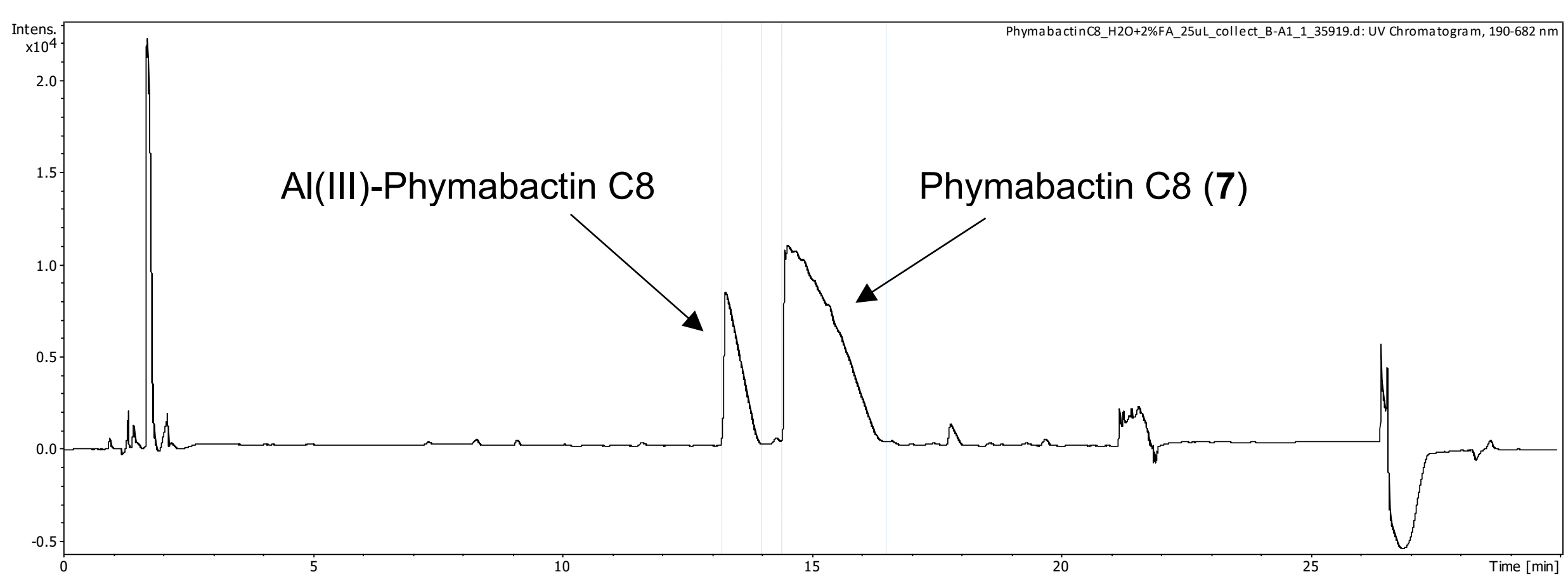

Fig. S3 Second purification step of **7** by analytical HPLC. The UV chromatogram with detection wavelengths of 190-682 nm is shown.

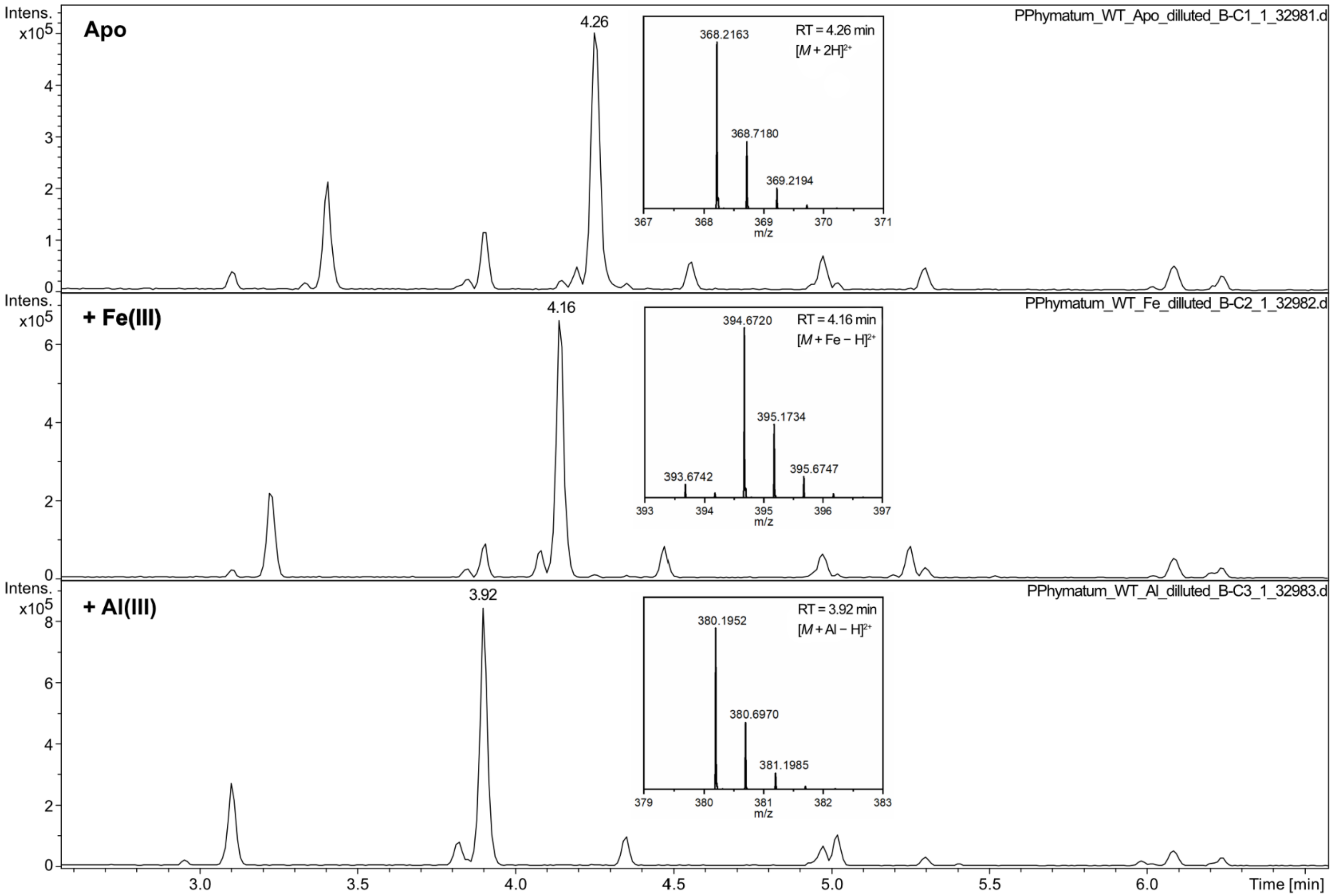

Fig. S4 Base peak chromatograms (BPC) obtained from siderophore screening of *P. phymatum* supernatant by UHPLC-HRMS. Complexation of apo-siderophores was carried out with $FeCl_3$ and $AlCl_3$. The mass spectra of phymabactin C8 (**7**) and its metal complexes are shown in the square boxes.

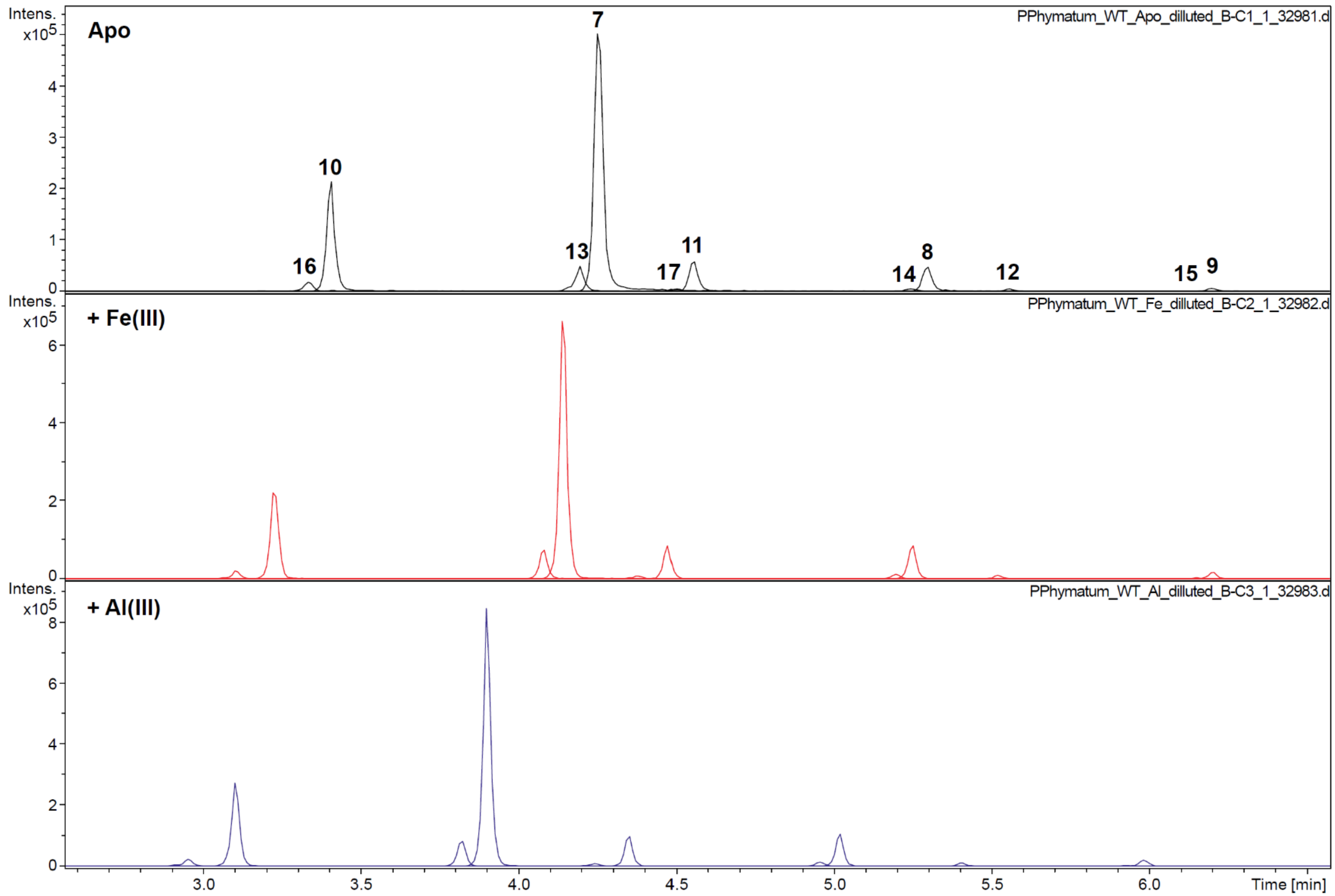

Fig. S5 Combined extracted ion chromatograms (EIC ± 0.01 Da) of phymabactin derivatives (**7–17**) obtained from siderophore screening of *P. phymatum* supernatant by UHPLC-HRMS. The apo-, iron- and aluminum-siderophores were detected as $[M + 2H]^{2+}$, $[M + Fe - H]^{2+}$ and $[M + Al - H]^{2+}$ ions, respectively.

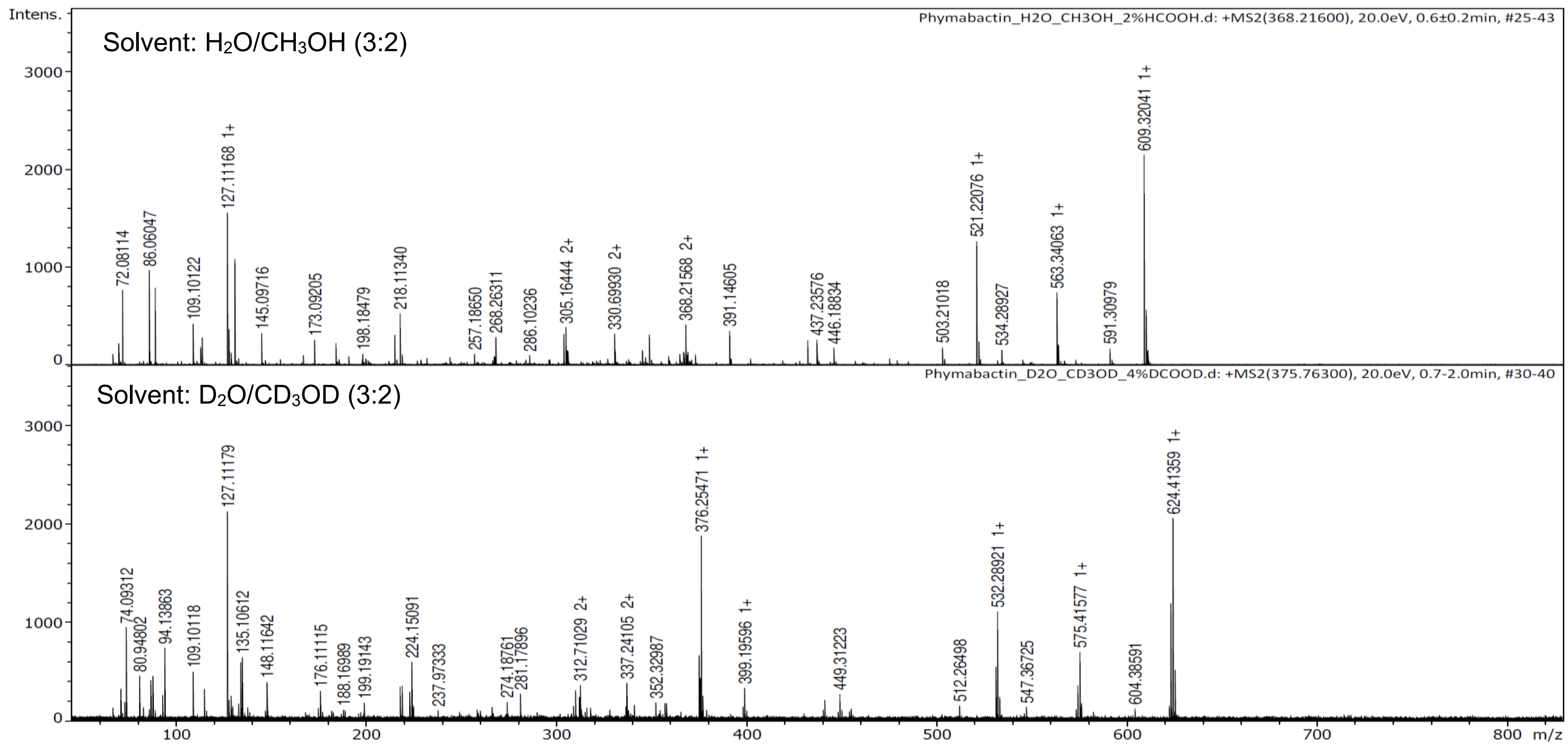

Fig. S6 MS/MS spectra of **7** dissolved in H_2O/CH_3OH (precursor: $[M + 2H]^{2+}$) and in D_2O/CD_3OD (precursor: $[d_{13}\text{-}M + 2D]^{2+}$). H/D-exchange of **7** indicates the presence of 13 exchangeable protons ($[d_{13}\text{-}M + 2D]^{2+}$ at *m/z* 375.76301). Both MS/MS spectra were recorded at a collision energy of 20 eV.

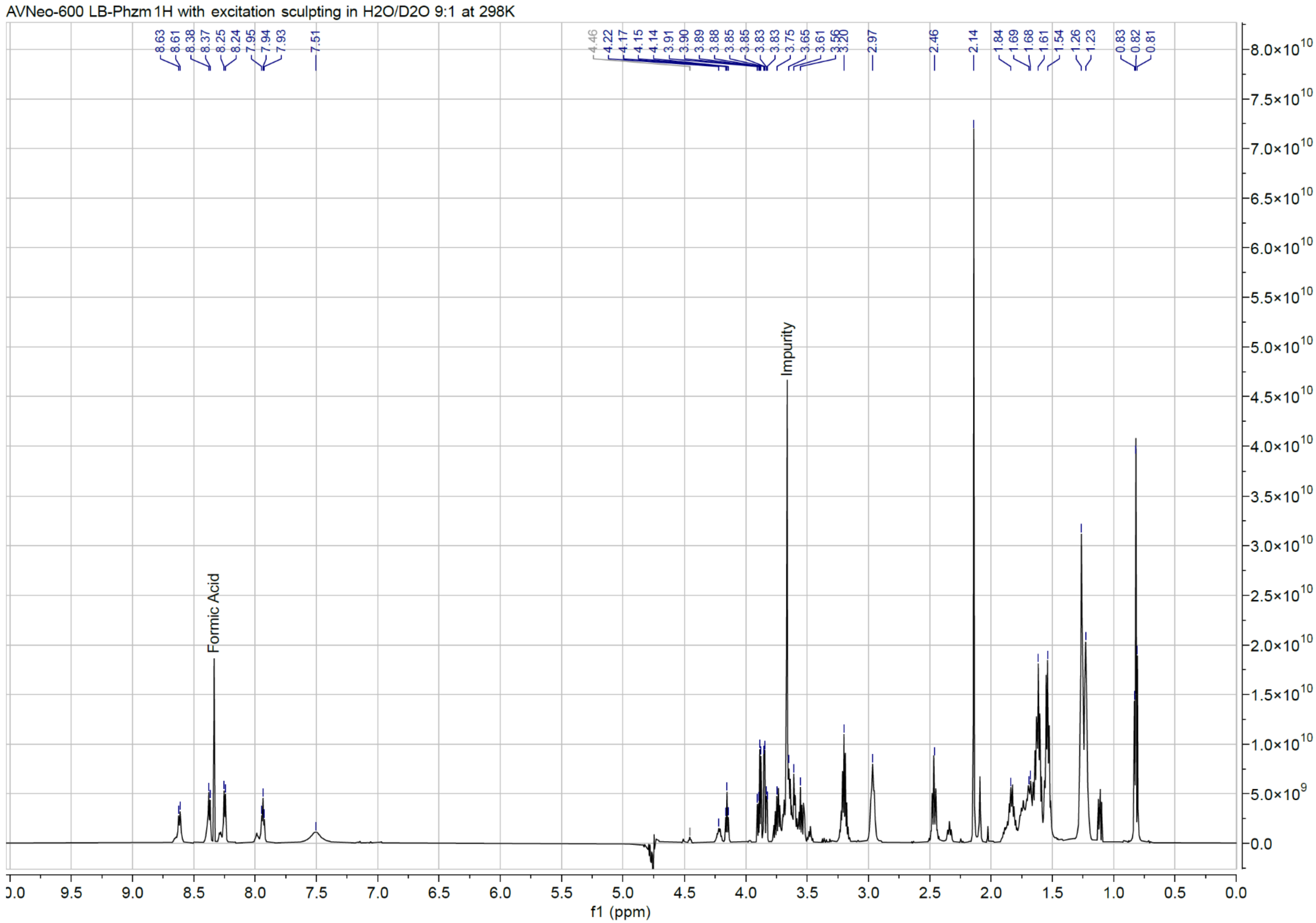

Fig. S7 ^{1}H NMR spectrum of **7**.

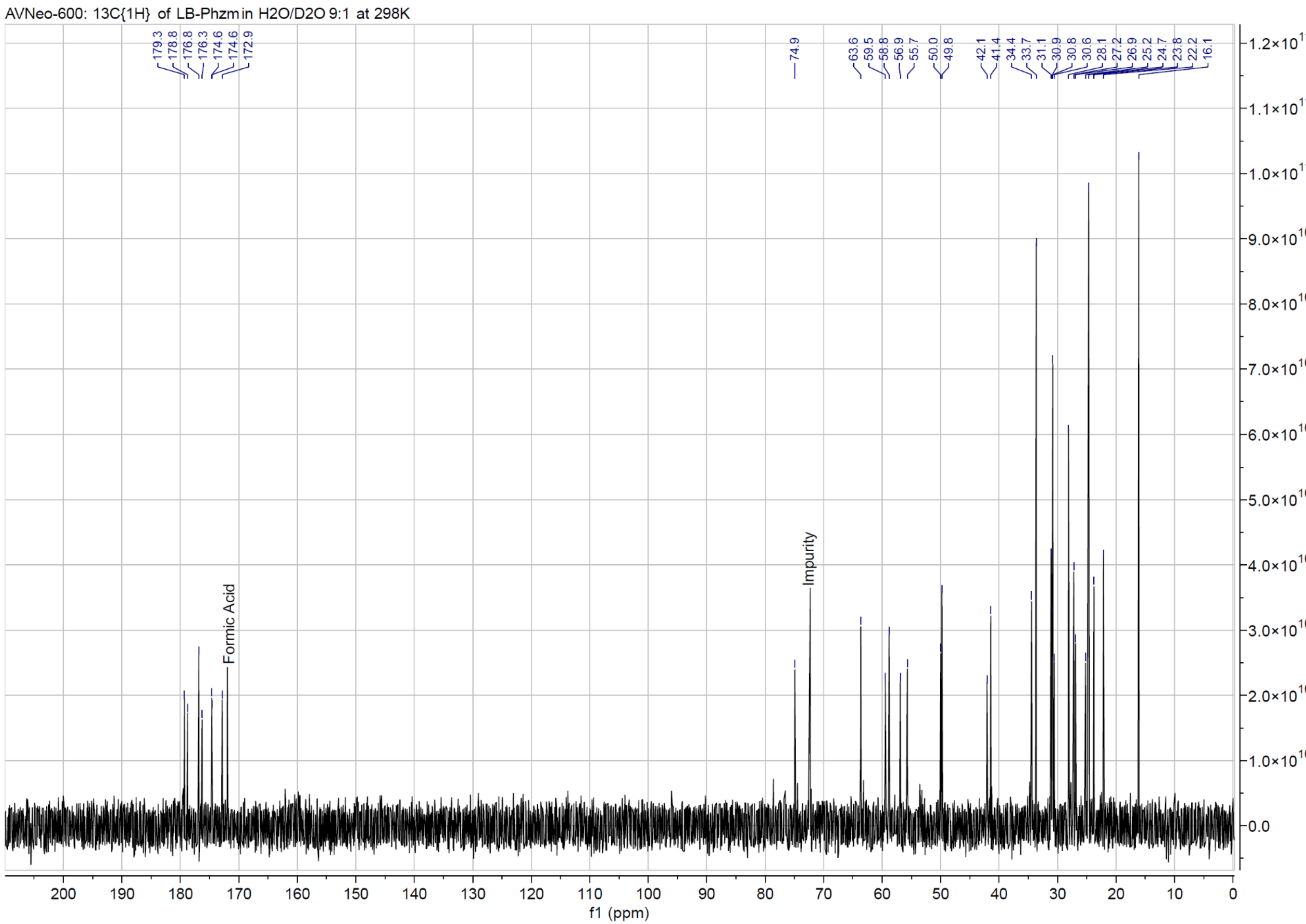

Fig. S8 ^{13}C NMR spectrum of **7**.

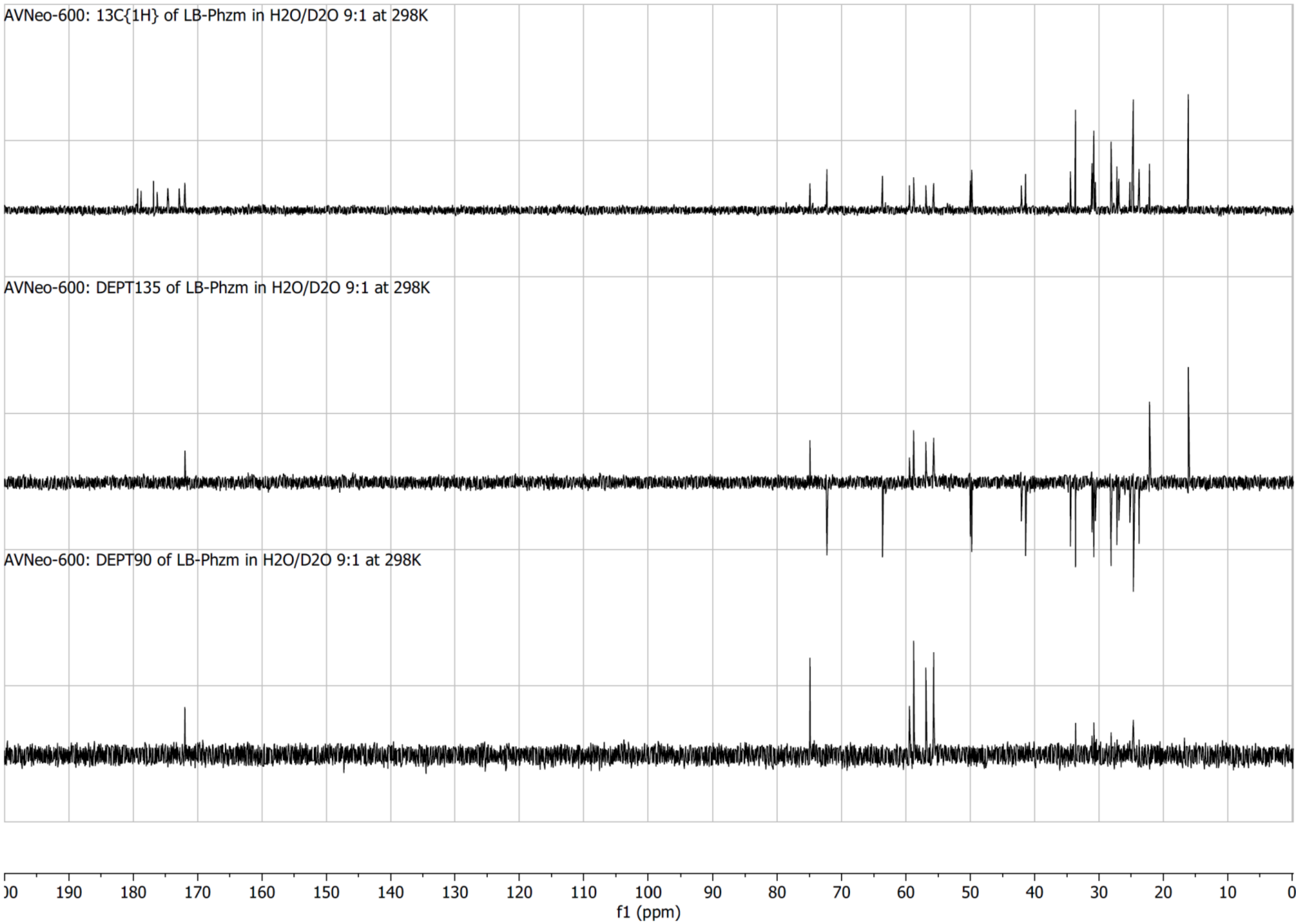

Fig. S9 ^{13}C, DEPT135 and DEPT90 NMR spectra of **7**.

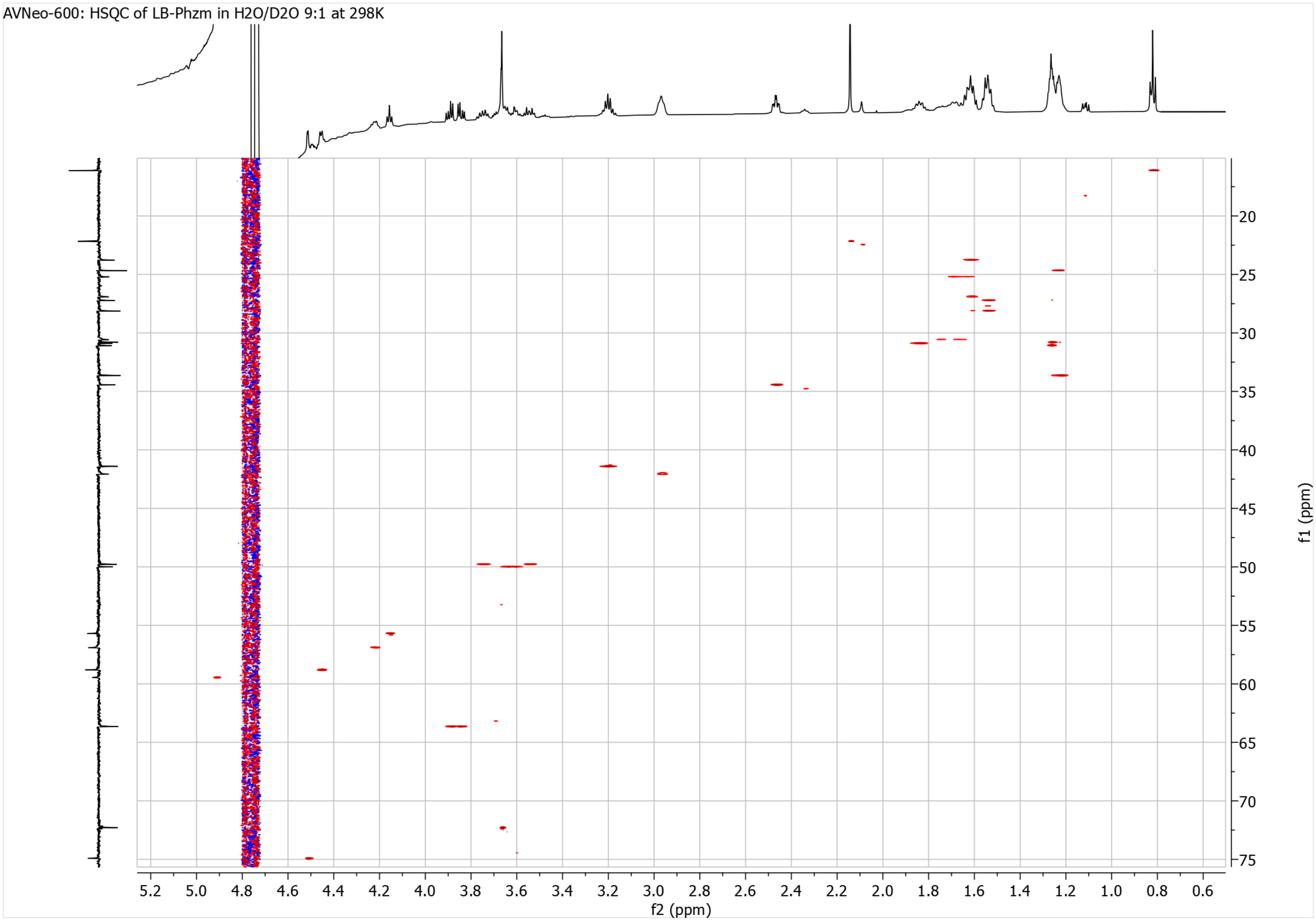

Fig. S10 HSQC NMR spectrum of **7**.

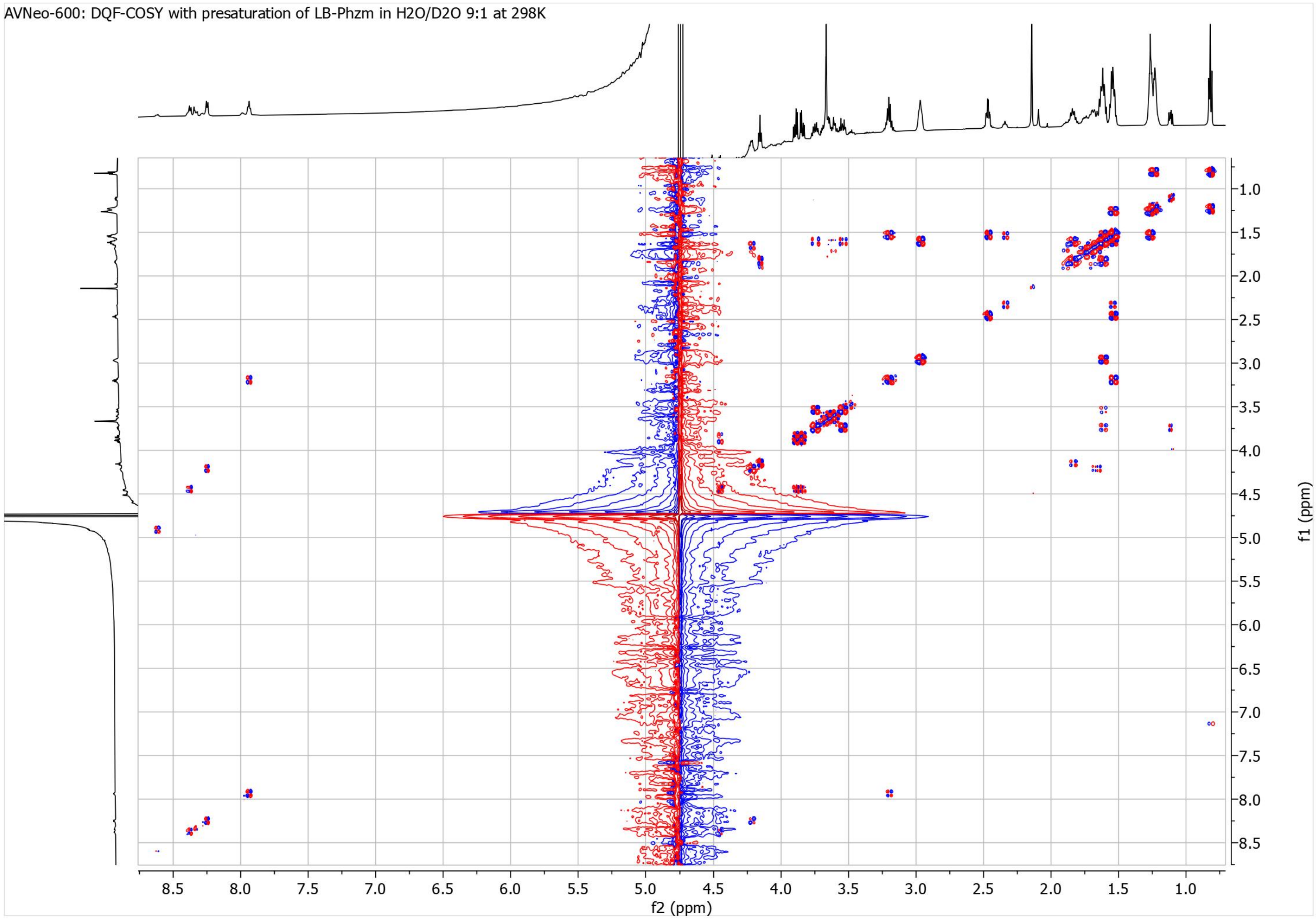

Fig. S11 DQF-COSY NMR spectrum of **7**.

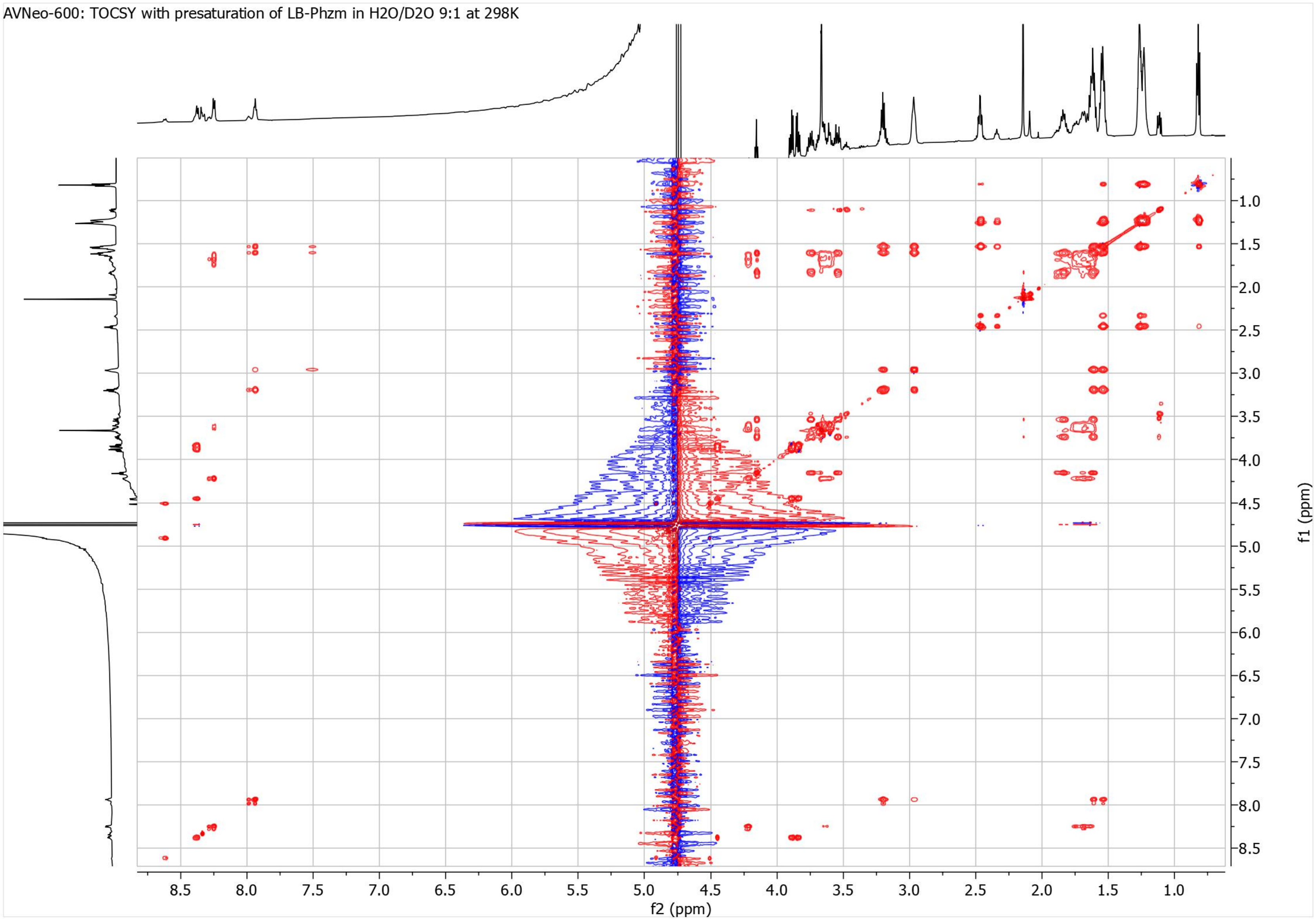

Fig. S12 TOCSY NMR spectrum of **7**.

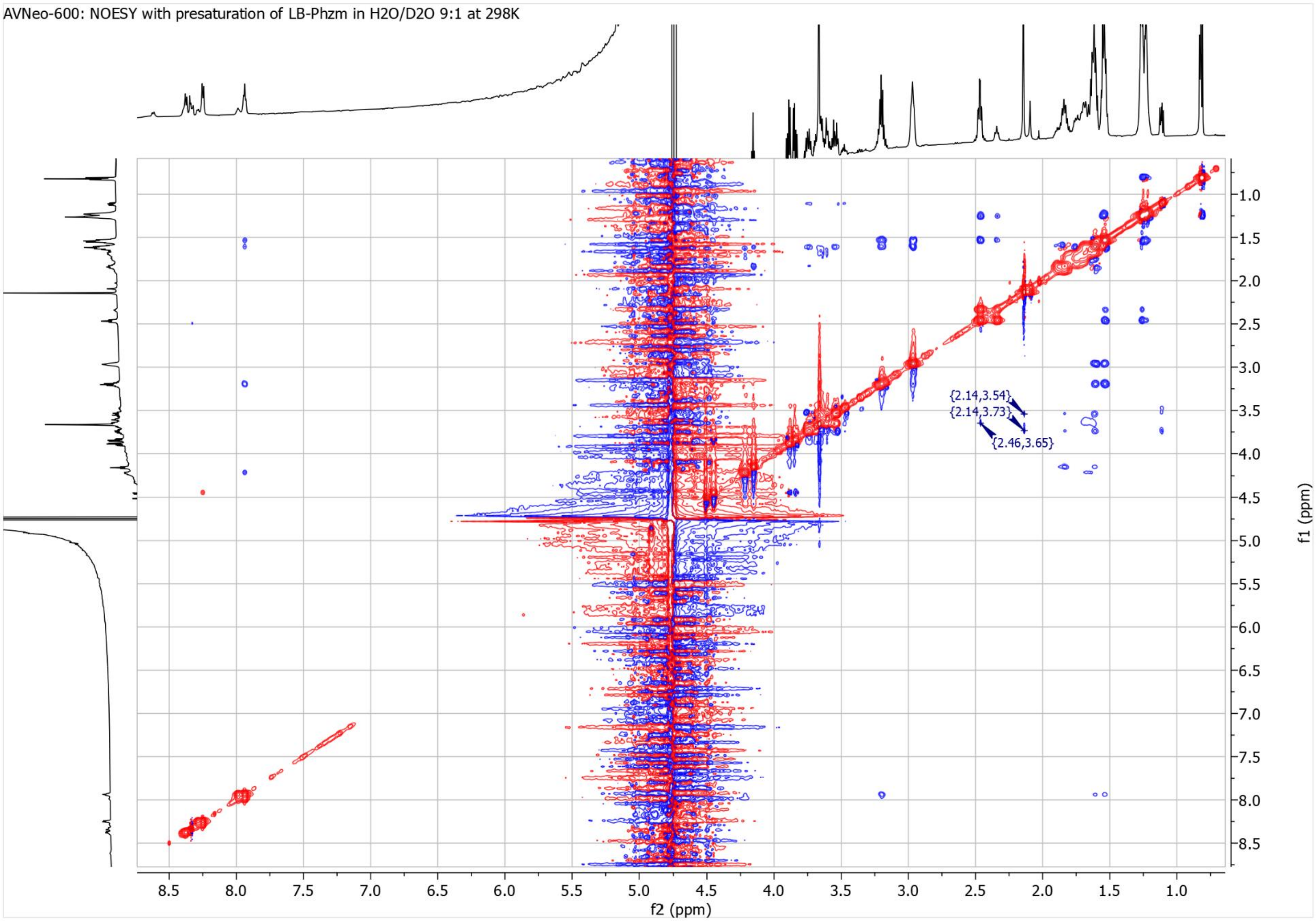

Fig. S13 NOESY NMR spectrum of **7** including peak labels for key correlations.

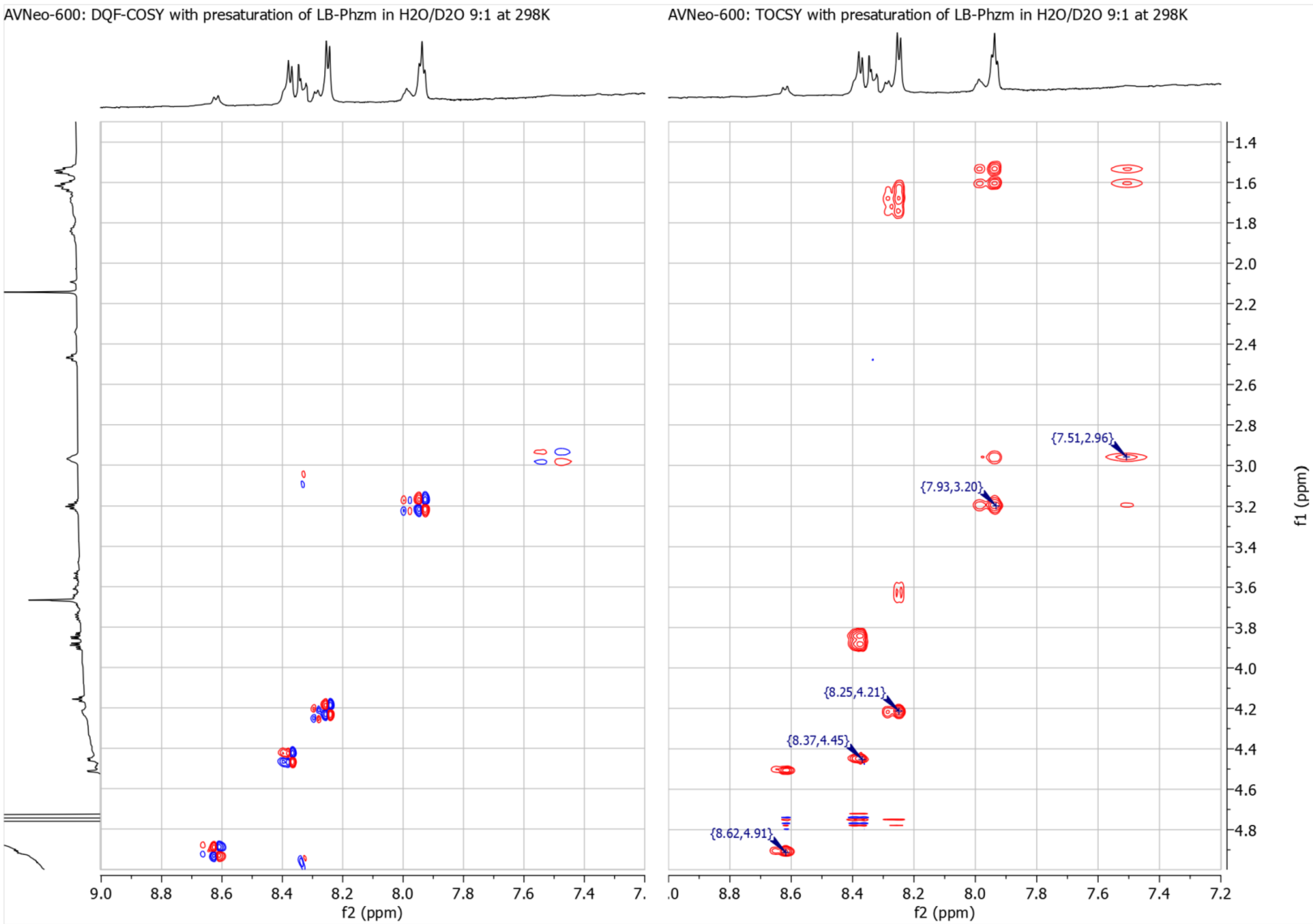

Fig. S14 Zoom into DQF-COSY (left) and TOCSY (right) NMR spectra of **7** including peak labels for key correlations.

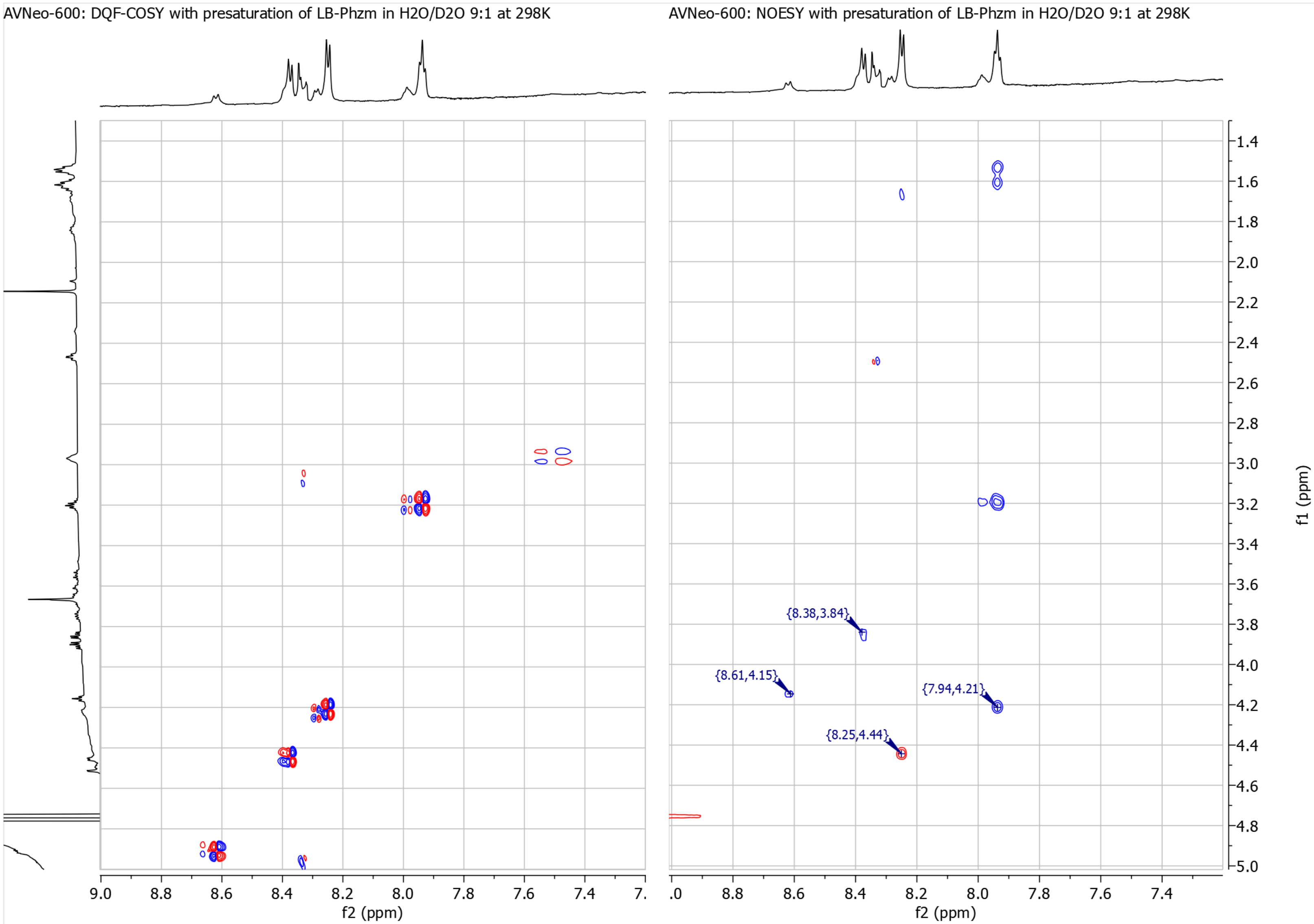

Fig. S15 Zoom into DQF-COSY (left) and NOESY (right) NMR spectra of **7** including peak labels for key correlations.

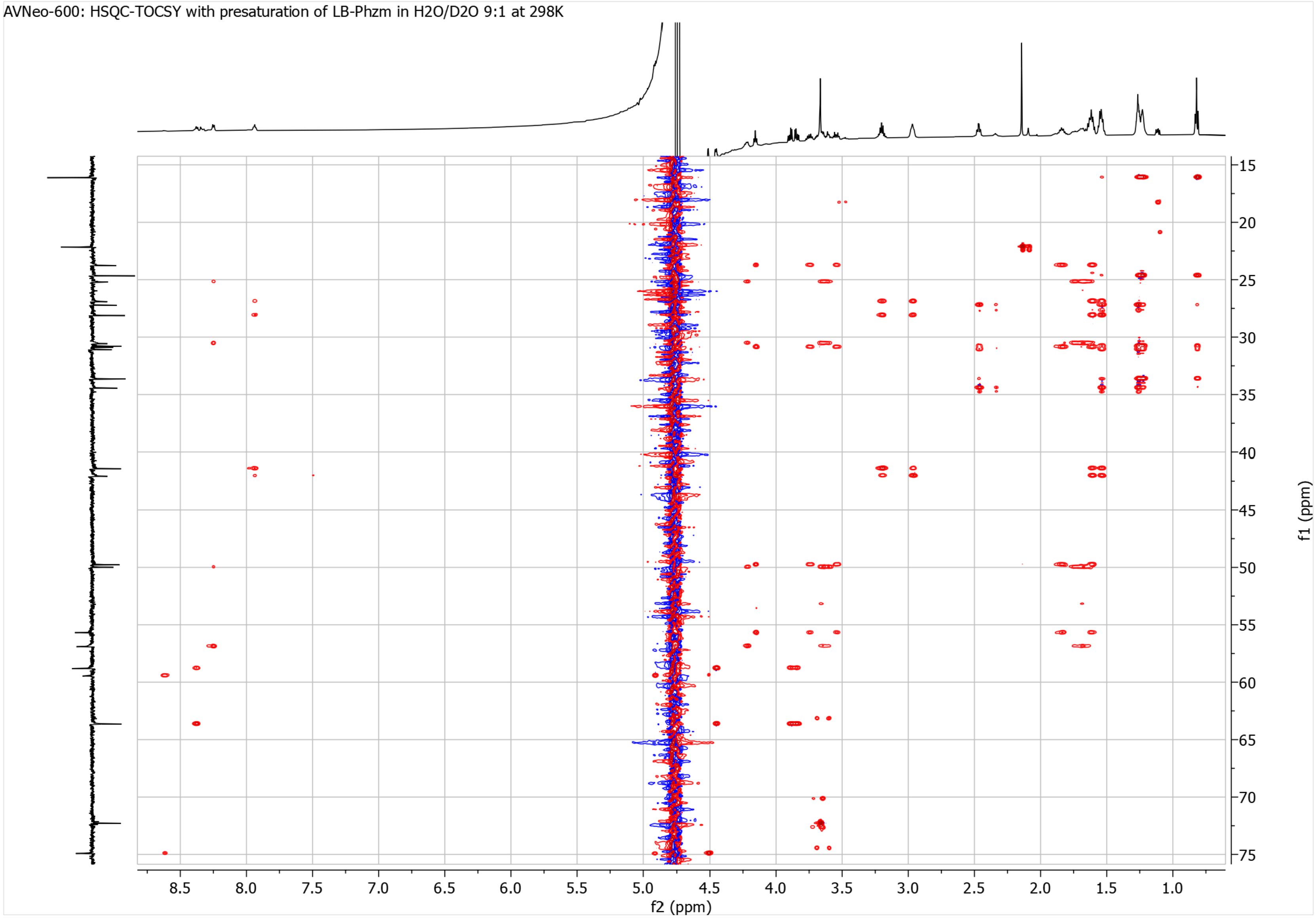

Fig. S16 HSQC-TOCSY NMR spectrum of **7**.

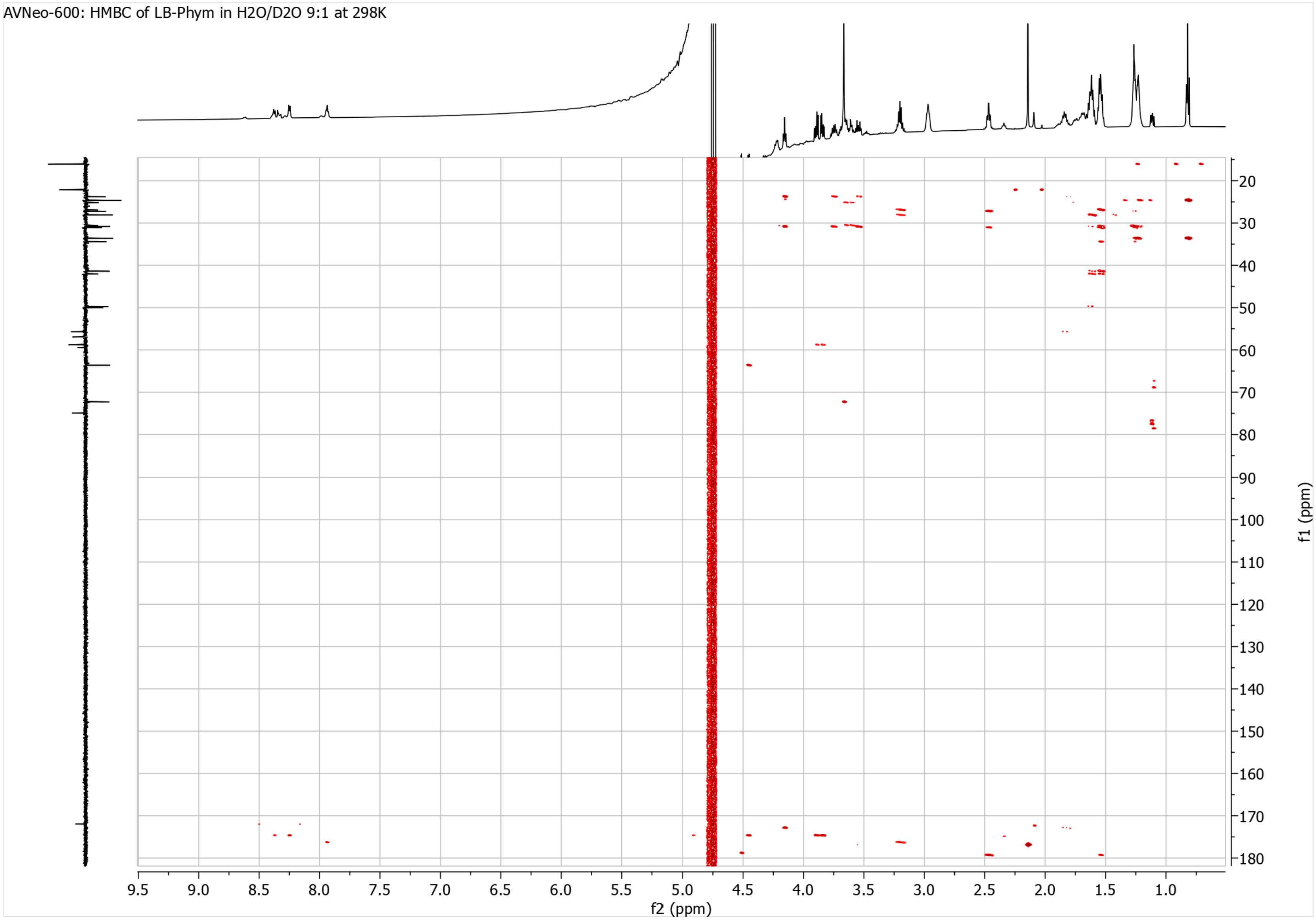

Fig. S17 HMBC NMR spectrum of **7**.

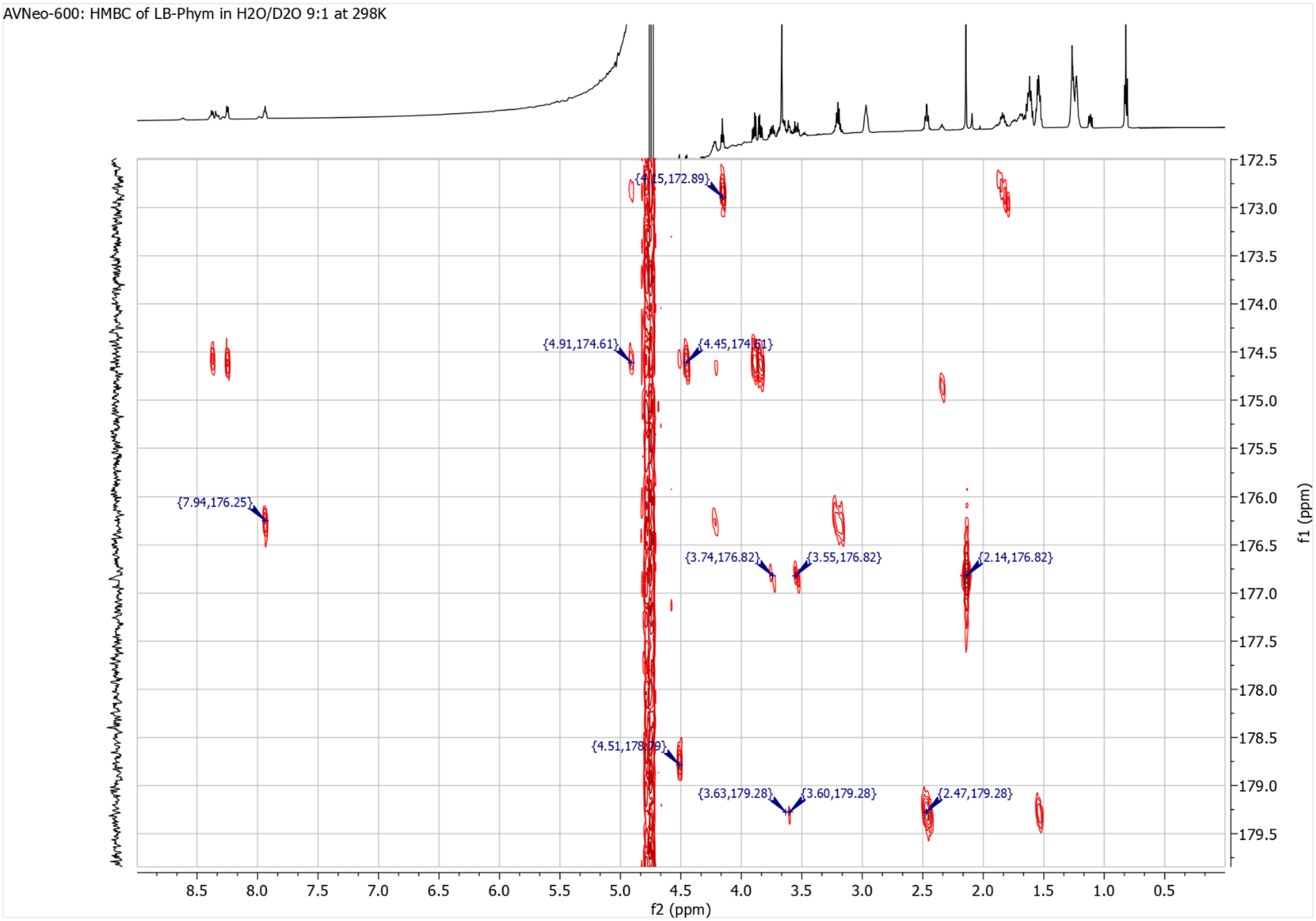

Fig. S18 Zoom into HMBC NMR spectrum of **7** including peak labels for key correlations.

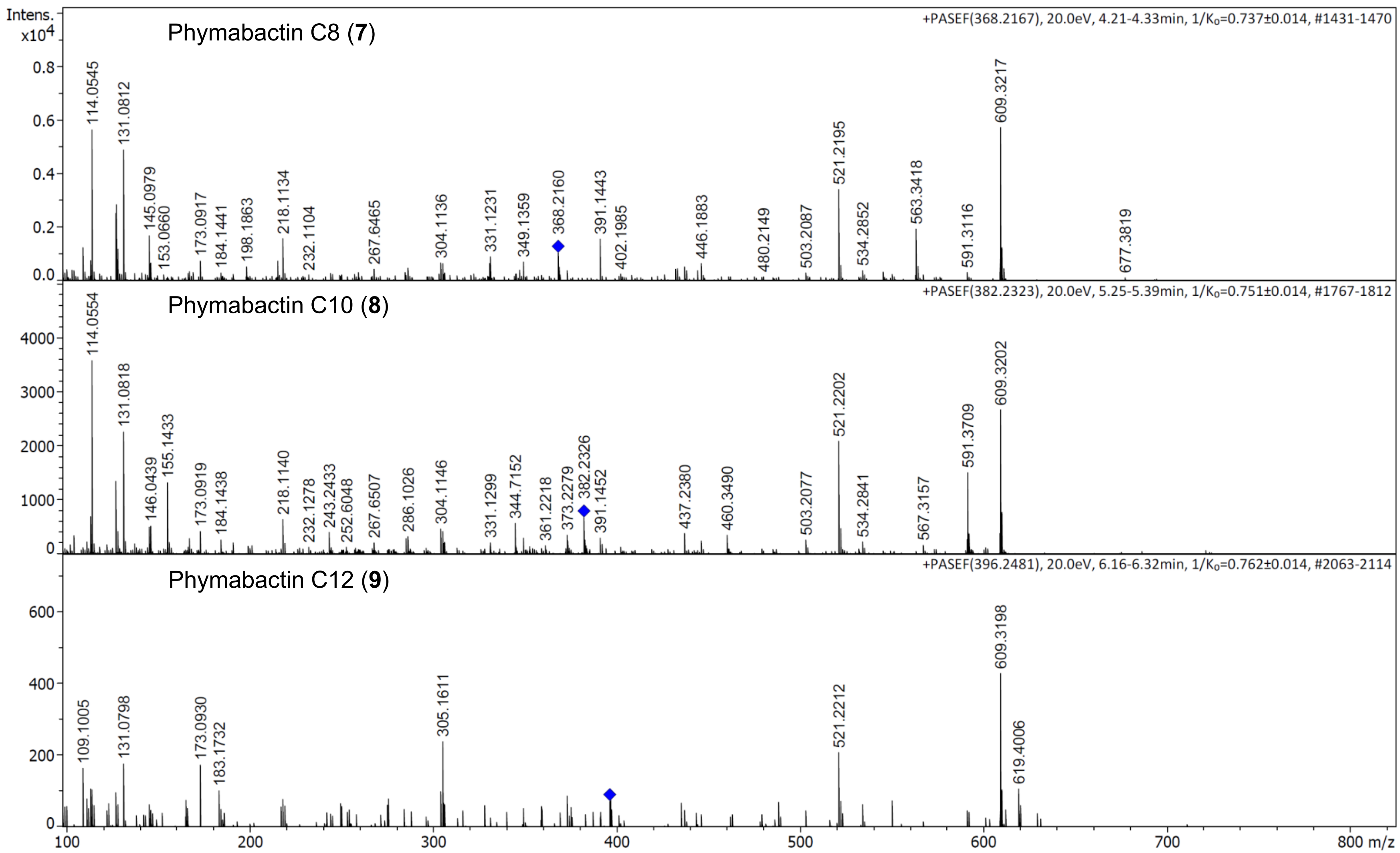

Fig. S19 MS/MS spectra of **7–9** acquired in the PASEF mode. Double-charged [*M* + 2H]$^{2+}$ precursor ions (blue squares) were fragmented at a stepped collision energy of 20 and 50 eV.

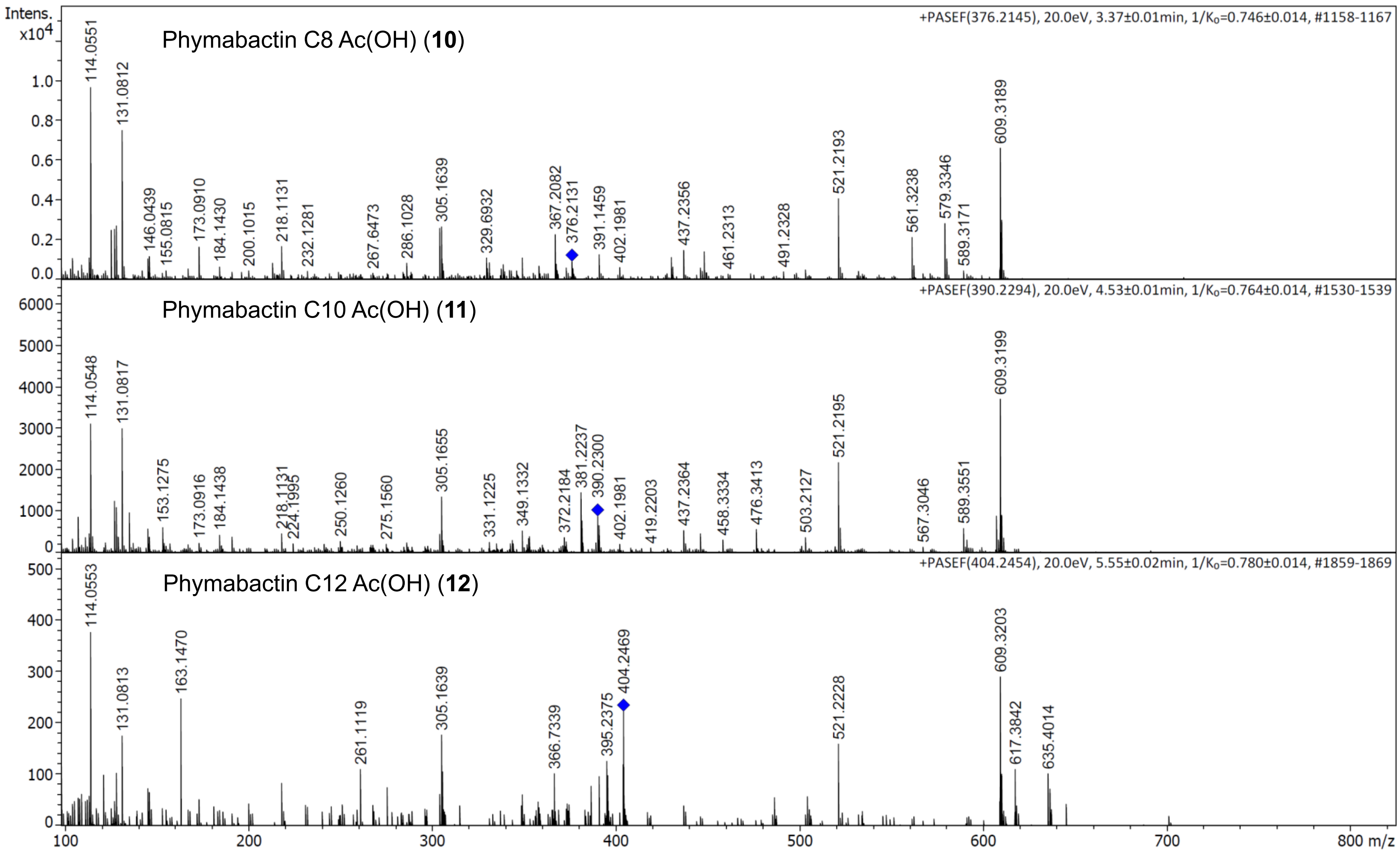

Fig. S20 MS/MS spectra of **10–12** acquired in the PASEF mode. Double-charged $[M + 2H]^{2+}$ precursor ions (blue squares) were fragmented at a stepped collision energy of 20 and 50 eV.

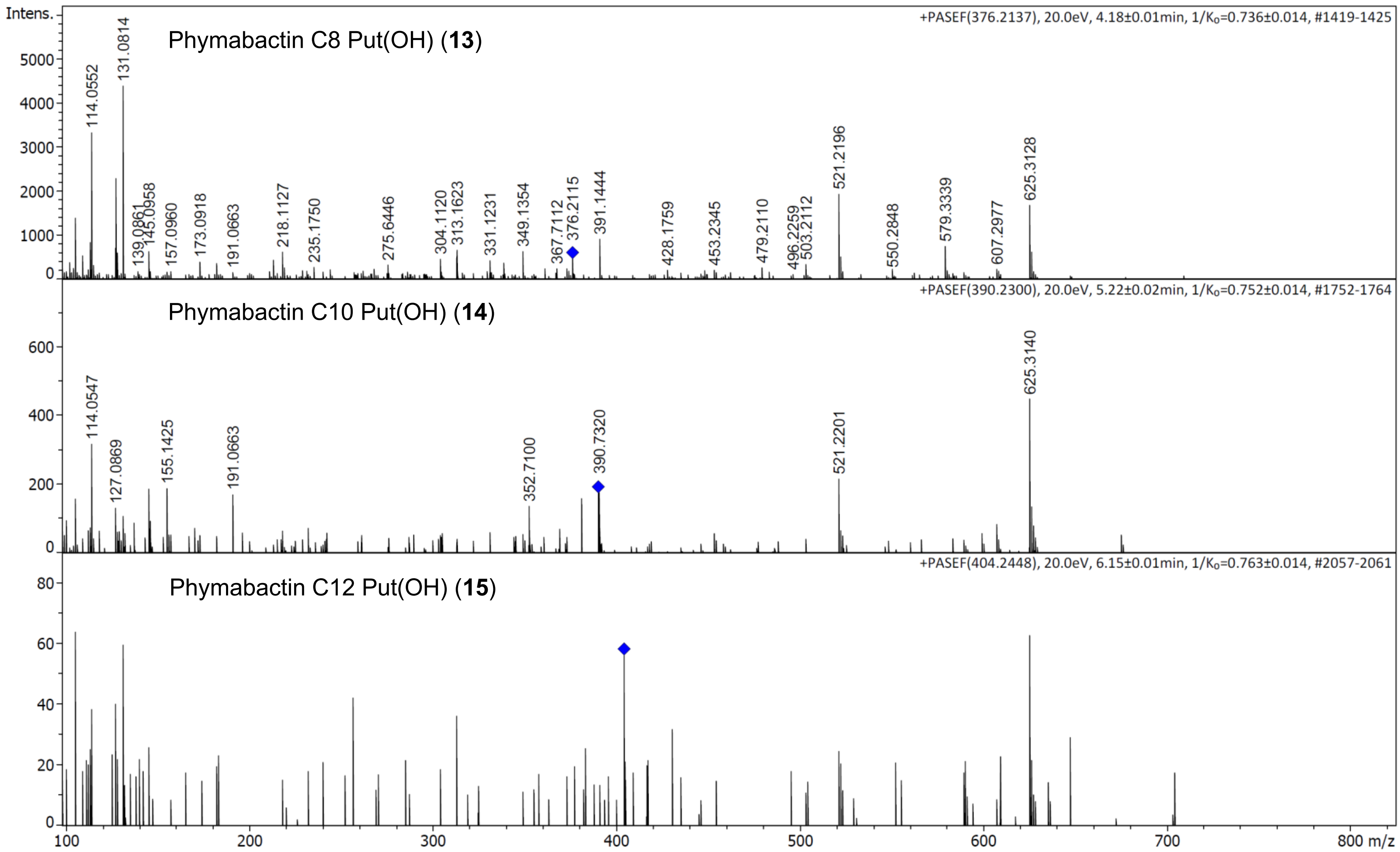

Fig. S21 MS/MS spectra of **13–15** acquired in the PASEF mode. Double-charged $[M + 2H]^{2+}$ precursor ions (blue squares) were fragmented at a stepped collision energy of 20 and 50 eV.

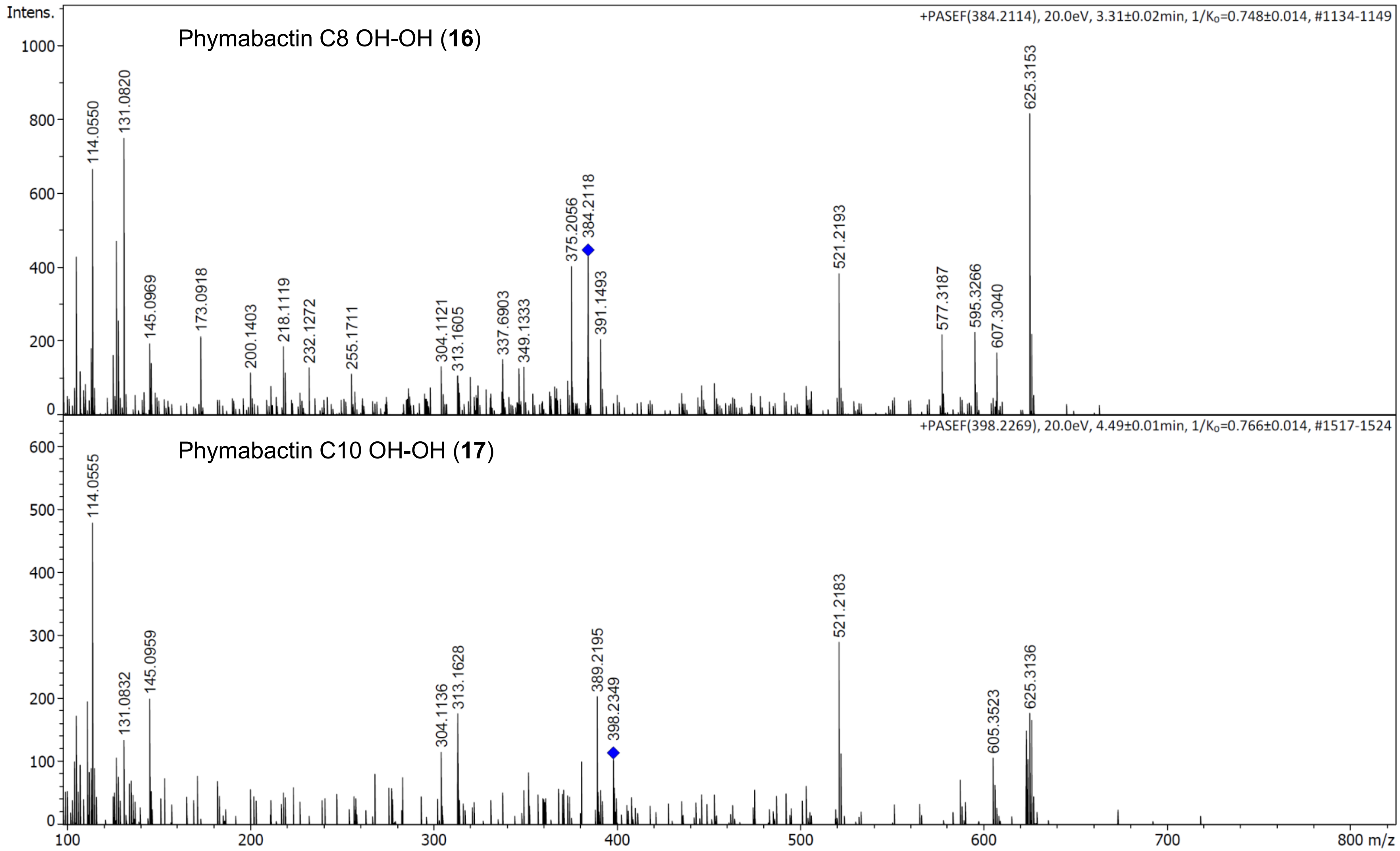

Fig. S22 MS/MS spectra of **16–17** acquired in the PASEF mode. Double-charged [*M* + 2H]$^{2+}$ precursor ions (blue squares) were fragmented at a stepped collision energy of 20 and 50 eV.

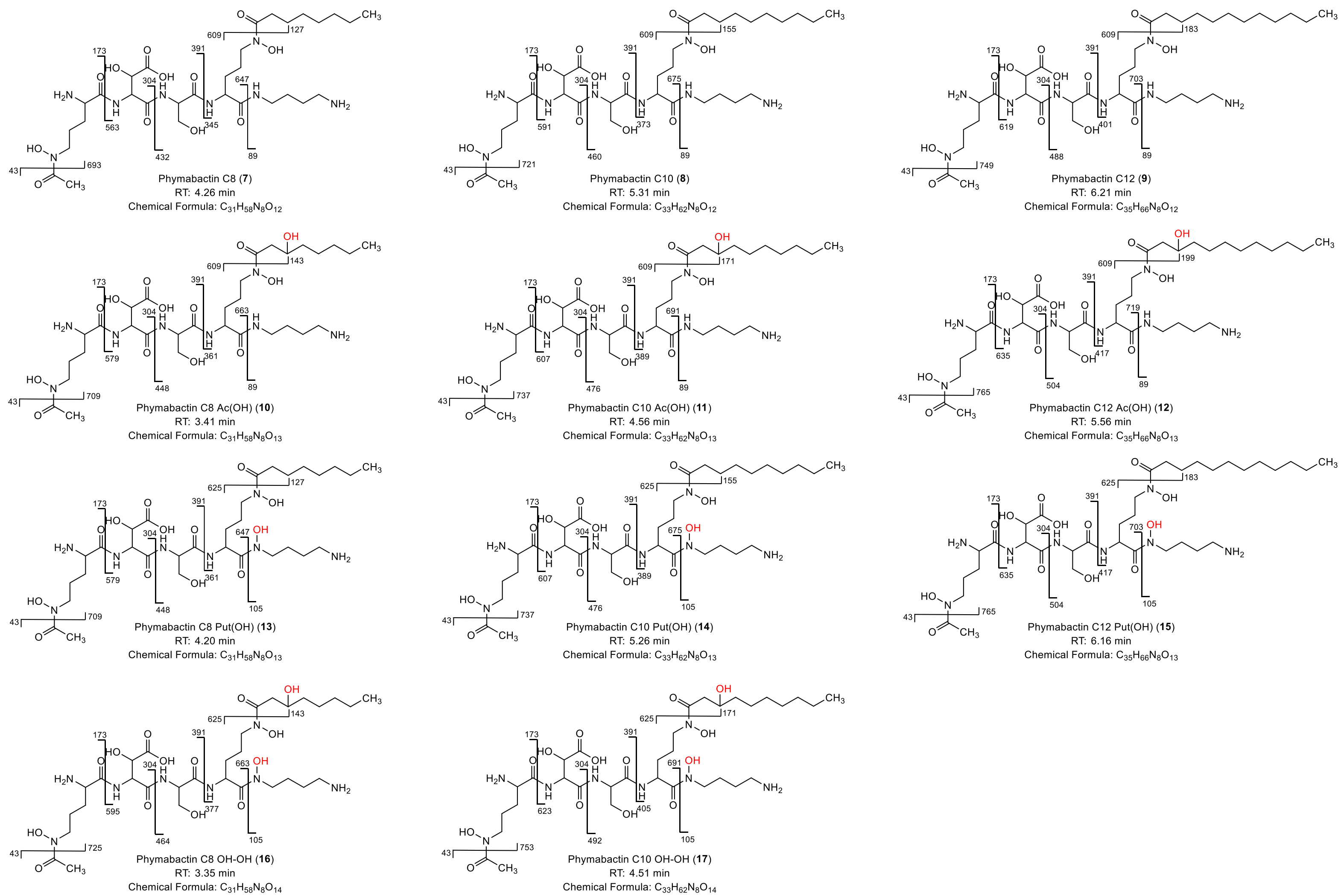

Fig. S23 Structural formulas of eleven phymabactin derivatives (**7–17**) including masses of expected MS/MS fragments, observed RT and chemical formulas. Oxidation was observed on acyl (Ac) and putrescine (Put) residues. The position of the red hydroxyl groups is not proven and was proposed in analogy to hydroxylated side chains that have been described in bacteria.
